# Western-style diet impedes colonization and clearance of *Citrobacter rodentium*

**DOI:** 10.1371/journal.ppat.1009497

**Published:** 2021-04-05

**Authors:** Junqing An, Xu Zhao, Yanling Wang, Juan Noriega, Andrew T. Gewirtz, Jun Zou

**Affiliations:** 1 Institute for Biomedical Sciences, Georgia State University, Atlanta, Georgia, United States of America; 2 Institute of Antibiotics, Huashan Hospital, Fudan University, Shanghai, China; University of California Davis School of Medicine, UNITED STATES

## Abstract

Western-style diet (WSD), which is high in fat and low in fiber, lacks nutrients to support gut microbiota. Consequently, WSD reduces microbiota density and promotes microbiota encroachment, potentially influencing colonization resistance, immune system readiness, and thus host defense against pathogenic bacteria. Here we examined the impact of WSD on infection and colitis in response to *Citrobacter rodentium*. We observed that, relative to mice consuming standard rodent grain-based chow (GBC), feeding WSD starkly altered the dynamics of *Citrobacter* infection, reducing initial colonization and inflammation but frequently resulting in persistent infection that associated with low-grade inflammation and insulin resistance. WSD’s reduction in initial Citrobacter virulence appeared to reflect that colons of GBC-fed mice contain microbiota metabolites, including short-chain fatty acids, especially acetate, that drive Citrobacter growth and virulence. Citrobacter persistence in WSD-fed mice reflected inability of resident microbiota to out-compete it from the gut lumen, likely reflecting the profound impacts of WSD on microbiota composition. These studies demonstrate potential of altering microbiota and their metabolites by diet to impact the course and consequence of infection following exposure to a gut pathogen.

## Author summary

Broad changes in societal dietary habits, especially consumption of processed foods that are frequently rich in fats and simple carbohydrates but lacking in fiber are widely believed to have contributed mightily to the increased incidence of chronic inflammatory diseases that has accompanied industrialization. Here we showed that dietary fiber results in a favorable colon environment that promotes *C*. *rodentium* growth and virulence. Consequently, a high-fat, low-fiber western-style diet reduced initial *C*. *rodentium* colonization but also resulted in a gut microbiota that was unable to mediate *C*. *rodentium* clearance from the colon. Persistent *C*. *rodentium* colonization associated with chronic low-grade inflammation and insulin resistance. Our study suggests that such pathogens, or perhaps even pathobionts like organisms such as *E*. *coli*, may not be readily cleared from hosts consuming high-fat low-fiber diets and thereby promote the chronic, often low-grade, inflammation related diseases.

## Introduction

The intestinal tract is densely colonized by a diverse group of microorganisms collectively referred to as gut microbiota [1]. A major benefit of gut microbiota is to protect its host from infection by bacterial pathogens [2]. Such protection is mediated by ability of microbiota to directly impede colonization of pathogens via niche occupation, production of bacteriocins, and consumption of essential nutrients, all of which are collectively termed colonization resistance [3]. Additionally, microbiota drives development, and regulates tone of, the mucosal immune system, which is critical to survival in response to challenge by many bacterial pathogens [4–6]. The importance of microbiota in defense against gut pathogens is readily appreciated by the stark increase in proneness to a variety of pathogens, including *Salmonella* and *C*. *difficile*, following antibiotic treatment [7, 8]. Use of a tractable model to study the role of the microbiota, namely use of germfree mice, also indicate a critical role of the microbiota in mediating clearance of gut pathogens from the intestinal lumen while host immunity cleared the pathogen from the epithelium [9, 10].

It is increasingly appreciated that, irrespective of antibiotics, gut microbiota composition is influenced by a variety of environmental (i.e. non-genetic) factors, especially diet [11, 12]. The majority of microbiota reside in the cecum and colon thus relying upon digestion-resistant complex carbohydrates, i.e. fiber, as a carbon source [13]. Furthermore, a variety of other dietary components are not completely absorbed and thus present in variable amounts that can modulate microbiota composition and function. Consequently, alteration of diet, can impact absolute numbers of gut bacteria (i.e. microbiota density) and influence its species composition, gene expression and localization [14–18]. Indeed, it has been proposed that broad changes in societal dietary habits, especially consumption of processed foods that are frequently rich in fats and simple carbohydrates but lacking in fiber may have impacted microbiota in a manner that promotes a variety of chronic inflammatory diseases such as inflammatory bowel disease, metabolic syndrome, and cancer [19–22]. While the nature of such changes are complex and multifactorial, we hypothesize that a well-nourished microbiota drives enterocyte proliferation, mucus secretion, and IL-22-mediated production of antimicrobial peptides, which collectively maintain a robust barrier that prevents microbiota encroachment, thereby avoiding inflammation [17]. Accordingly, we postulate that reduced nourishment of microbiota, particularly due to lack of dietary fiber, has contributed to the post mid-20^th^ century increased prevalence of chronic inflammatory diseases such as inflammatory bowel disease, metabolic syndrome, and cancer.

Ability of diet to impact microbiota and, consequently, host immunity and colonocyte metabolism can also be envisaged to impact outcomes following encounters with gut pathogens[23, 24]. For example, the reduction in microbiota density resulting from a low-fiber diet might reduce colonization resistance and impede host defense, including reduced production of mucus and antimicrobial peptides. Furthermore, lack of fiber results in microbiota digesting mucus further weakening its ability to impede pathogens [13]. On the other hand, one might imagine that starving microbiota via lack of fiber, might limit availability of nutrients required by pathogens thus impeding pathogens colonization. Additionally, alterations of the colonic environment that result from consumption of different diets might influence virulence gene expression, and thus extent of disease, caused by gut pathogens. Moreover, altered fiber content is but one change in dietary habits that might impact host-pathogen interactions. Indeed, reduced consumption of dietary fiber is often associated with increased consumption of fat and simple carbohydrates. Hence, the central goal of this study was to investigate how change from a relatively unrefined grain-based chow (GBC) diet to a highly processed high-fat western style diet (WSD) impacted infection with a well-studied murine pathogen, namely *Citrobacter rodentium*. We observed that the colonic environment in WSD-fed mice impeded its initial colonization but also failed to support microbiota-mediated clearance.

## Results

### WSD feeding alters gut microbiota composition and dynamics of C. rodentium infection

**Fig 1:**
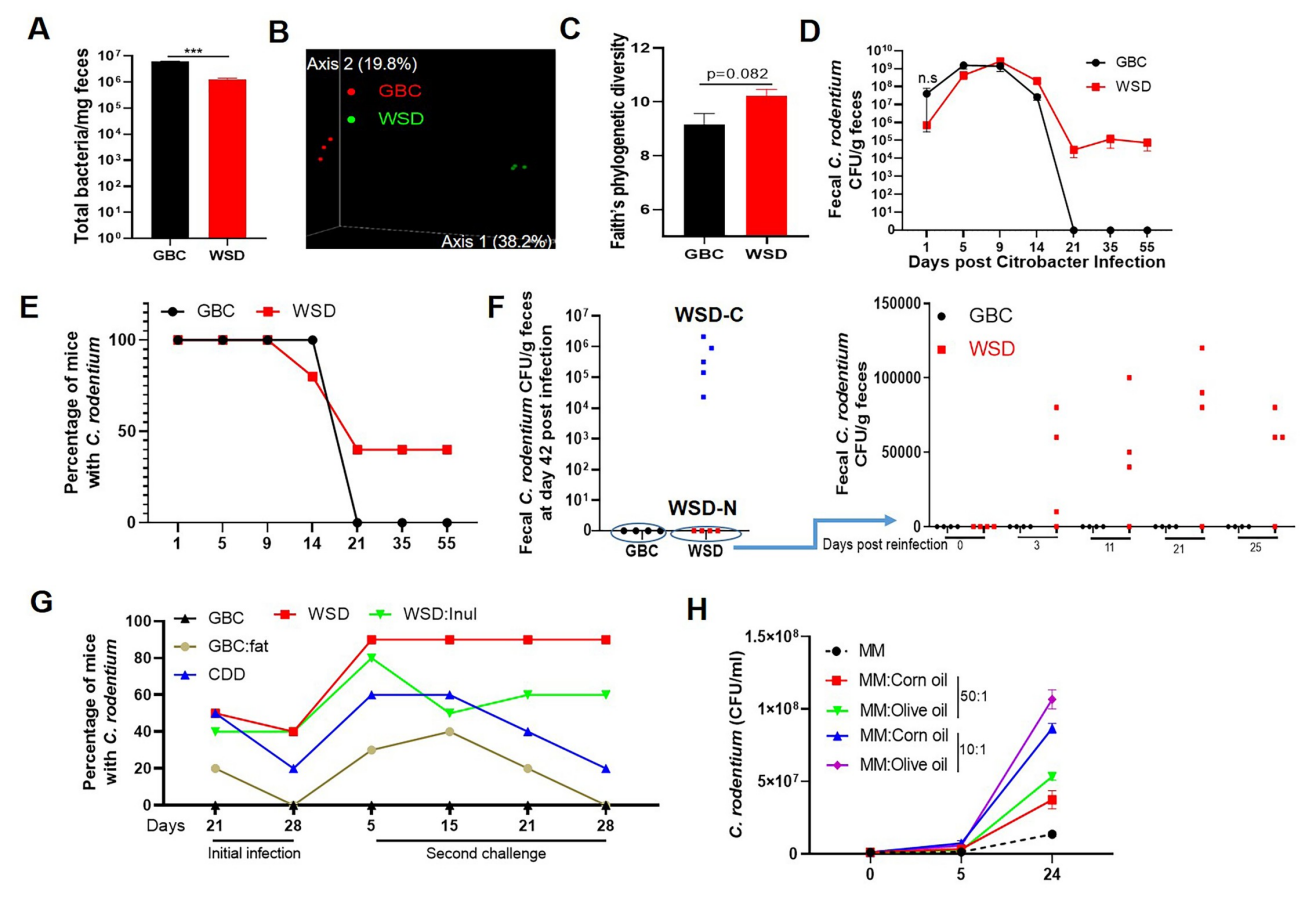
WSD-induced alterations in gut microbiota associate with inability to clear *C*. *rodentium*. **A-C.** Adult C57BL/6 mice were maintained on GBC or administered WSD for 1 week. **A.** Bacteria per mg feces were measured by qPCR. **B.** Gut microbiota composition was analyzed by 16s rRNA sequencing followed by UniFrac PCoA analysis. **C.** Faith’s phylogenetic diversity. **D-E.** Fecal *C*. *rodentium* CFU following its oral inoculation. Mean fecal CFU per mouse (**D**) and percentage of mice with detectable *C*. *rodentium* (**E**). **F.** Mice that had no detectable *C*. *rodentium* (GBC or WSD-N) were re-inoculated with *C*. *rodentium*, and fecal *C*. *rodentium* CFU quantitated. **G**. Ability of mice fed indicated diets to clear primary and secondary *C*. *rodentium* challenge was assayed as in E-F. **H**. Growth curves of *C*. *rodentium* in minimal media supplemented with different types/amount of lipid. Data are the means +/- SEM (n = 3–10 mice per group) of an individual experiment and representative of 2–3 separate experiments.

Consistent with our previous work [17], switching mice from a standard grain-based rodent chow (GBC) to a high-fat low-fiber compositionally defined western-style diet (WSD) resulted in a rapid reduction in the total number of bacteria per mg feces (Fig 1A), which was associated with altered gut microbiota composition as indicated by Principal Coordinates Analysis (PCoA, Fig 1B) and taxonomic analysis (S1A and S1B Fig), including increased alpha diversity as measured by Faith’s phylogenic diversity (Fig 1C). To investigate if such changes impacted proneness to a gut pathogen, mice were orally challenged with 5x10^8^ colony forming units of *Citrobacter rodentium*. Relative to GBC-fed mice, those fed WSD displayed a modest non-statistically significant reduction in fecal *C*. *rodentium* particularly at early timepoints (Fig 1D); day 1 p = 0.44 by 2-tailed Mann-Whitney test. Yet, the most striking consequence of WSD consumption was frequent inability of mice to clear this pathogen (Fig 1D and 1E). Specifically, *C*. *rodentium* was uniformly absent in the feces of GBC-fed mice by 21 d post-inoculation but remained present in 40% of mice consuming WSD for the additional 5 weeks that they were monitored. Furthermore, while GBC-fed mice were completely resistant to subsequent challenge with *C*. *rodentium*, those WSD-fed mice that had cleared *C*. *rodentium* were prone to developing chronic infection when re-challenged by this pathogen (Fig 1F).

One primary difference between GBC and WSD is their 5% vs. 25% respective fat content (wt/wt). Thus, to specifically examine the role of such high-fat content, we re-proportioned the semi-purified ingredients to approximate the fat content of GBC (**S1 Table)**. Consumption of this low-fat compositionally defined diet (CDD) recapitulated the delayed clearance of *C*. *rodentium* and, moreover, proneness to re-infection although most CDD-fed mice eventually cleared *C*. *rodentium* (Figs 1G and S1C). The WSD *C*. *rodentium* persistence phenotype was partially mimicked by enriching GBC with the same lipid used in formulating WSD (GBC: fat) (Figs 1G and S1C). In vitro studies demonstrated *C*. *rodentium* had capacity to utilize polyunsaturated and monounsaturated fat, corn and olive oil respectively, as sole carbon sources (Fig 1H). Together, these results indicate that high-fat content contributes to, but is not sufficient to fully recapitulate, the WSD *C*. *rodentium* persistence phenotype. Another key feature of WSD is its low fiber content (5% by wt) and complete lack of fermentable fiber, whereas GBC is comprised of about 20% fiber, including similar amounts of fermentable and non-fermentable (insoluble) fiber. Enriching WSD with a fermentable fiber, inulin, restores many aspects of host-microbiota homeostasis, including bacterial density, and prevents WSD-induced metabolic syndrome [17, 25]. However, enriching WSD with inulin (WSD:inul) did not significantly aid clearance of *C*. *rodentium* indicating that reduced bacterial density was not sufficient to explain the inability of WSD-fed mice to clear *C*. *rodentium* (Figs 1G and S1C).

### Low virulence of *C*. *rodentium* in WSD mice

**Fig 2:**
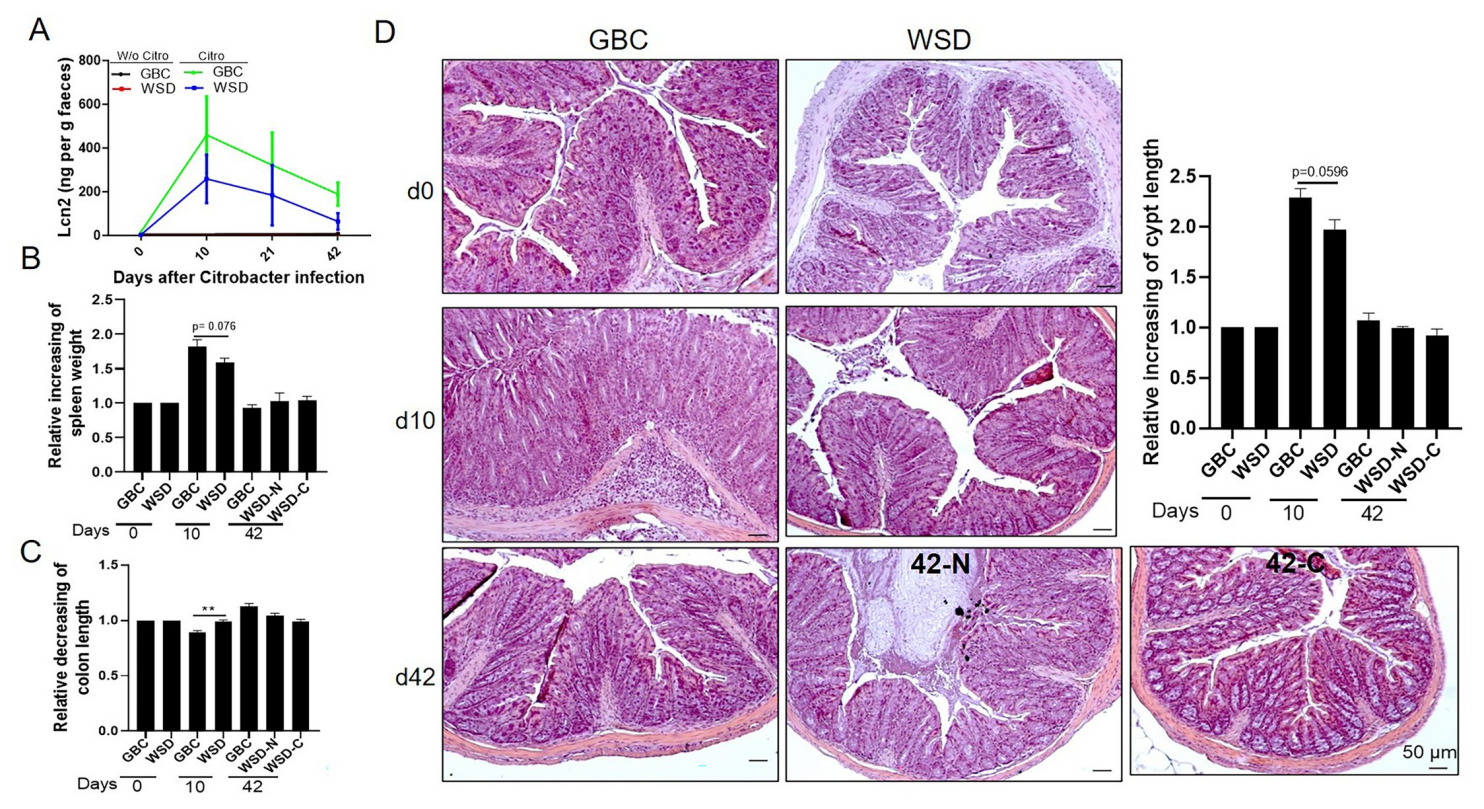
Reduced acute inflammation and lack of overt chronic inflammation in WSD-fed mice challenged with *C*. *rodentium*. C57BL/6 mice were administered PBS or *C*. *rodentium* and euthanized at the indicated time points. **A.** Measurement of fecal lipocalin 2 by ELISA. **B-C**. Spleen weight and colon length was measured in GBC and WSD fed mice that had (WSD-C) or lacked (WSD-N) detectable fecal *C*. *rodentium* CFU, and the relative fold change of spleen and colon were normalized to day 0 values of mice fed with the respective diet. **D-E**. Colon tissue was processed for H&E staining, which afforded measure of colon crypt length, and expressed as relative fold changes to day 0 values of mice fed with the respective diet. Data are the means +/- SEM (n = 5–10 mice per group) of an individual experiment and representative of 2–3 separate experiments.

**Fig 3:**
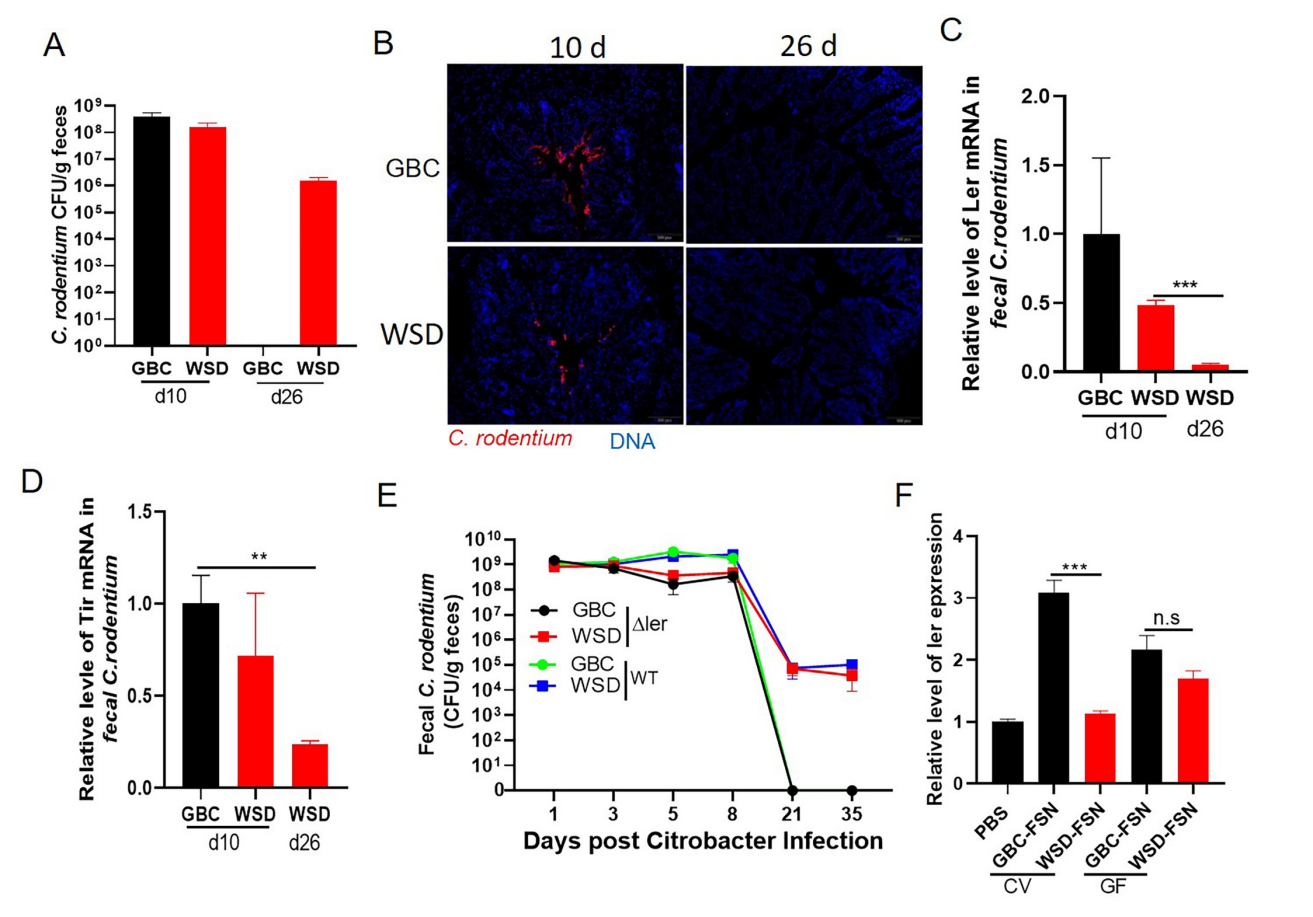
WSD feeding resulted in reduced *C*. *rodentium* virulence gene expression. **A**. Quantification of fecal *C*. *rodentium* at the indicated days post-inoculation. **B**. Immunofluorescent staining of *C*. *rodentium* in colon tissue at the indicated days post-inoculation. **C-D**. The relative expression level of *C*. *rodentium* virulence genes measured by qRT-PCR. **E**. GBC and WSD fed mice were orally gavaged with kanamycin and, 16h later, inoculated with WT and Δ*ler C*. rodentium. Fecal *C*. *rodentium* CFU was monitored. **F.** Ler expression of *C*. *rodentium* grown in minimal media supplemented with fecal supernatant (FSN) from conventional (CV) or germ-free (GF) mice as indicated by a luminescent *ler* reporter strain. Results were expressed as the relative fold changes of luminescence by normalizing to CFU of ler-lux *C*. *rodentium* in the same samples. Data are the means +/- SEM (n = 3–5 per group) and representative of 2 separate experiments.

Next, we examined the extent to which WSD influenced *C*. *rodentium*-induced disease. Mice consuming GBC or WSD were euthanized 10d following *C*. *rodentium* inoculation, at which time levels of *C*. *rodentium* were similar between the 2 diets, or 42d, at which time only some WSD-fed mice remained infected with *C*. *rodentium*. Levels of the fecal inflammatory marker lipocalin-2 (Lcn2) peaked at 10d in both groups and were moderately lower 10–42 days post-inoculation in WSD-fed mice (Fig 2A). Accordingly, WSD-fed mice exhibited modest reduced indications of acute inflammation as reflected by splenomegaly (Fig 2B), colon shortening (Fig 2C), colonic hyperplasia (crypt lengthening) (Fig 2D) and depletion of goblet cells (S2 Fig), all of which are typical features of *C*. *rodentium* colitis. Moreover, the moderate degree of gut inflammation observed in WSD-fed mice resolved with similar kinetics as that of GBC-fed mice. Irrespective of whether *C*. *rodentium* persisted, at 42 d post infection, WSD-fed mice lacked abnormal mucosal pathology such as goblet cell depletion, which is often observed in chronic infection (Figs 2 and S2) thus suggesting the persisting *C*. *rodentium* may lack virulence gene expression. In accord, mucosal-adherent *C*. *rodentium* was present at 10d, but not 26d in mice fed either diet (Fig 3A and 3B). Moreover, the persisting *C*. *rodentium* displayed low expression of key virulence genes, namely *ler* and *tir*, normalized for levels of *C*. *rodentium*, relative to d10 feces (Fig 3C and 3D). To test the notion that the d26 persisting *C*. *rodentium* was functionally low in virulence, feces from WSD-fed (d10 and d26) and GBC-fed (d10) mice were transferred to immune deficient mice, Rag2^-/-^IL2Rg^-/-^, which, unlike WT mice, can be readily infected by the relatively low *C*. *rodentium* inoculums (10^7^ CFU) achievable in fecal suspensions generated from d26 WSD-fed mice. Relative to d10 samples, transfer of d26 samples resulted in only modest levels of *C*. *rodentium* in the new host, further supporting the notion that the persisting *C*. *rodentium* was not highly virulent (S3A Fig). This result suggested such genes were not required for *C*. *rodentium* to persist in WSD-fed mice. We examined this possibility via use of a *C*. *rodentium* engineered to lack *ler*, which is known to be essential to colonize the mouse intestinal tract[9]. Such inability to colonize mice was not altered by WSD feeding (no CFU detected in feces of WSD-or GBC-fed mice inoculated with 5x10^8^ CFU *C*. *rodentium* Δ*ler*). However, colonization could be readily attained if mice were pre-treated with kanamycin. In this case, Δ*ler* mutants grew robustly in mice fed either diet to levels similar to its WT isogenic parent at early time points post-inoculation (Fig 3E). However, while *C*. *rodentium* was uniformly cleared from GBC-fed mice, WT and Δ*ler* remained detectable at similar levels in WSD-fed mice, indicating that *C*. *rodentium* persistence in WSD-fed mice does not depend upon its major virulence locus. The association of reduced virulence gene expression and inflammation on d10 in WSD-fed mice led us to hypothesize that WSD feeding would not reduce d10 inflammation in mice colonized with Δ*ler C*. *rodentium*. However, this strain did not result in any indices of inflammation in mice fed either diet (S3C–S3E Fig) thus making this approach relatively uninformative.

To further investigate the impact of diet on *C*. *rodentium* virulence gene expression, we measured how fecal supernatants (FSN) of GBC- and WSD-fed mice would impact *ler* expression in vitro via a bioluminescent reporter strain. Enriching minimal media with FSN from mice fed GBC, but not WSD, enhanced *ler* expression (Fig 3F). In contrast, the extent to which FSN isolated from germfree mice promoted *ler* expression was not significantly reduced by WSD feeding (Fig 3F). These results indicate the colonic environment that results when commensal microbiota is nourished by GBC is relatively favorable to *C*. *rodentium* virulence.

### Persistent *C*. *rodentium* infection promotes insulin resistance

**Fig 4:**
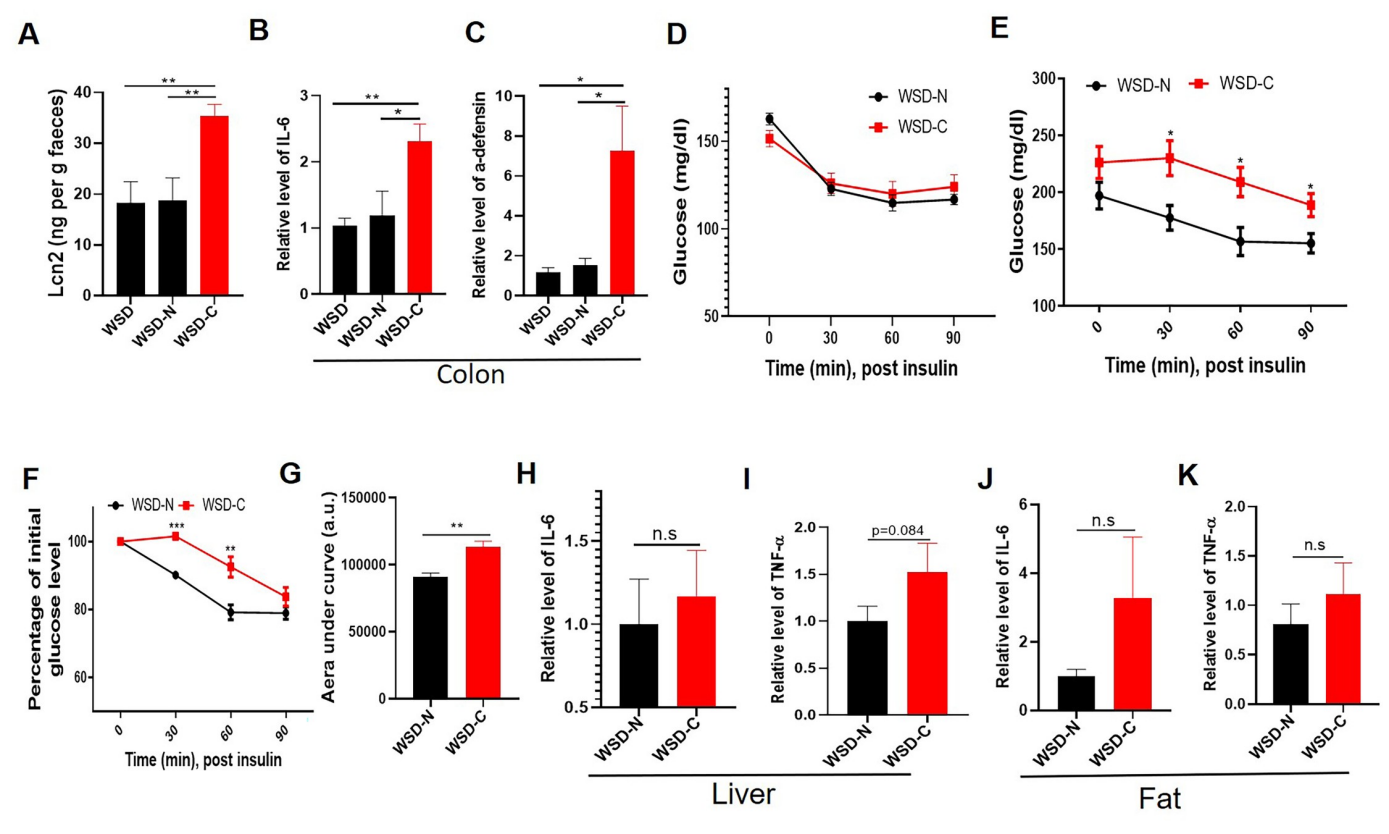
Persistence of Citrobacter infection in WSD-fed mice associates with low-grade inflammation and insulin resistance. **A-D**. Uninfected WSD-fed mice (WSD) and WSD-fed mice with (WSD-N) or without (WSD-C) C. rodentium clearance were assayed at 42d post- infection for fecal lipocalin 2 (Lcn2) by ELISA (A), colonic expression of IL-6 (B) and α-defensin (C) by qRT-PCR, and glycemic responses to insulin (D). **E-K**. Five months later, glycemic responses and indices of low-grade systemic inflammation were measured. Measure of absolute (E) and relative (F) glucose levels following insulin as well as area under curve were calculated (G). Relative level of IL-6, TNF-α in liver and epididymal fat were analyzed by qPT-PCR (H-K). Data are the means +/- SEM (n = 5 per group).

It is increasingly appreciated that some host-microbial interactions that do not cause overt, i.e. histopathologically evident, inflammation can promote pro-inflammatory gene expression that promotes insulin resistance, especially in the context of WSD-induced obesity [26]. Hence, we compared gene expression and metabolic parameters in WSD-fed mice that had, or had not, cleared *C*. *rodentium*. At 42 d, *C*. *rodentium* persistence associated with higher levels of lipocalin-2 and increased expression of inflammation-related genes in the colon (Fig 4A–4C) but not insulin resistance (Fig 4D). However, glycemic assessment of the mice 5 months post-inoculation found increased fasting blood glucose levels and reduced responsiveness to insulin in mice with persisting *C*. *rodentium* (Fig 4E–4G) despite similar weight and adiposity (S4A–S4C Fig). Such insulin resistance was associated with modest, not statistically significantly higher expression of canonical markers of low-grade inflammation, namely IL-6 and TNFα, in adipose tissue and liver (Fig 4H–4K). The association of chronic *C*. *rodentium* infection and dysglycemia suggested a potential role for persistent *C*. *rodentium* infection in exacerbating WSD-induced dysglycemia and/or that exacerbated dysglycemia that might occur in some mice impeded clearance of *C*. *rodentium*. To help differentiate these non-mutually exclusive possibilities, we examined if streptozotocin (STZ)-induced diabetes impacted the course of *C*. *rodentium* infection. We selected mice in which STZ elevated fasting glucose to levels only slightly higher than those of *C*. *rodentium*-infected WSD-fed mice (S5A Fig). Such hyperglycemia enhanced *C*. *rodentium* infection as described previously [27], and associated with delayed but nonetheless eventual complete clearance of the pathogen (S5B Fig). These results suggest that dysglycemia contributes to, but does not fully account for, the impaired clearance of *C*. *rodentium* in WSD-fed mice.

We next examined the extent to which persistent *C*. *rodentium* in WSD-fed mice might reflect impaired mucosal immunity. WSD, by itself, results in lower expression of IL-22, which is known to play an important role in protection against *C*. *rodentium*. However, IL-22 expression was comparable between GBC- and WSD-fed mice on d10 and was higher in mice persistently with C. rodentium than in GBC-fed mice that had cleared this pathogen at day 42 (S6A Fig). Analogously, WSD-fed mice displayed elevated levels of fecal and serum antibodies to *C*. *rodentium* arguing against the notion that defective adaptive immunity under *C*. *rodentium* accounted for the persistent infection (S6B and S6C Fig).

### WSD-induced changes in microbiota contribute to *C*. *rodentium* persistence

**Fig 5:**
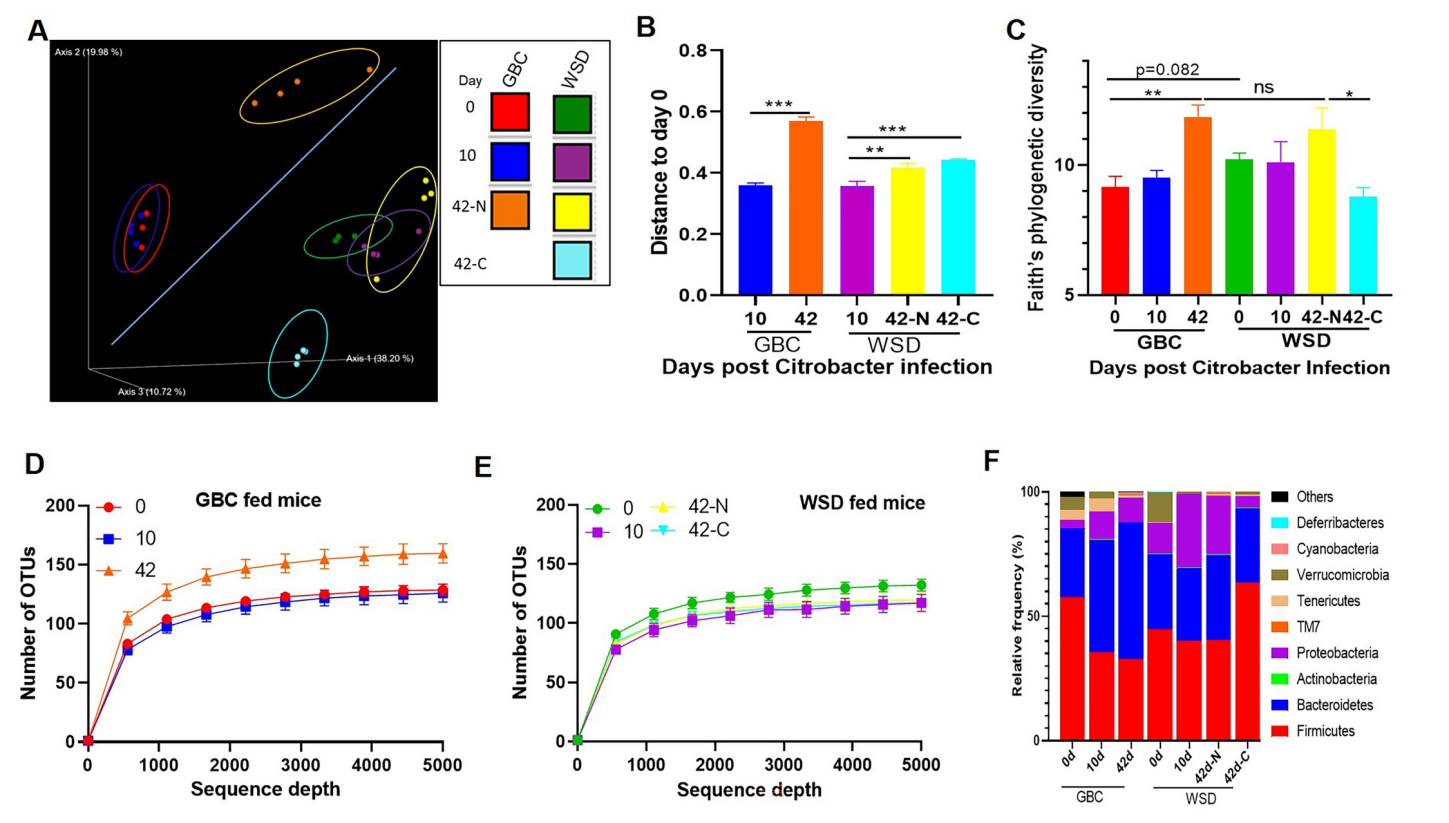
*C*. *rodentium* starkly alters microbiota of mice fed GBC but not WSD. **A-B**. Analysis of gut microbiota composition based on 16s rRNA sequencing and analyzed/displayed by unweighted UniFrac PCoA (A), UniFrac distances between Day 10 or 42 after *C*. *rodentium* infection relative to Day 0 (B). Alpha-diversity as measured by Faith’s PD (C) and observed OTUs (D&E). **F**. Relative abundance of bacteria at the phylum level.

While adaptive immunity, especially humoral immunity [28], mediates clearance of virulence gene-expressing *C*. *rodentium* from the epithelium, eliminating it from the outer mucus and lumen reflects the pathogen being out-competed by commensal microbiota [9]. Hence, we hypothesized that the inability of WSD-fed mice to eliminate *C*. *rodentium* might reflect differences in basal or *C*. *rodentium*-induced differences gut microbiota. One stark difference in basal microbiota in GBC- and WSD-fed mice is microbiota density. While this difference was moderately accentuated following *C*. *rodentium* administration (S7A Fig), restoring this parameter by enriching WSD with inulin did not reduce *C*. *rodentium* persistence, arguing against it having a major role in mediating *C*. *rodentium* clearance. Hence, we turned our attention toward microbiota composition. Assay of fecal microbiota by 16S sequencing, followed by PCoA and linear discriminant analysis effect size (LefSe) analysis revealed that the extent and duration of *C*. *rodentium*-induced changes microbiota composition was quite different between mice fed GBC and WSD. Specifically, while GBC-fed mice displayed a stark change in microbiota composition at d42, at which time they had uniformly cleared the pathogen, WSD-fed mice showed only modest changes at this time regardless of *C*. *rodentium* persistence (Figs 5A and 5B and S7B–S7E). Moreover, alpha-diversity, which was assessed by Faith’s phylogenetic diversity and number of observed taxonomic units (OTU), revealed increased community richness following *C*. *rodentium* administration in mice fed GBC but not WSD (Fig 5C–5E). Analysis at phylum and major genera levels revealed that mice fed WSD and GBC for 1 week had minimal Enterobacteriaceae before *C*. *rodentium* infection, and the percentage of Proteobacteria and Verrucomicrobia was increased in mice fed with WSD for 1 week. Changes in microbiota composition that associated with *C*. *rodentium* infection, in mice fed either diet, included increased abundance of *Proteobacteria*, and a decrease in the relative abundance of Verrucomicrobia (Fig 5F).

To consider how such changes in microbiota might impact *C*. *rodentium*, we utilized PICRUST to identify metabolic pathways associated with altered microbiota composition. Prior to exposure to *C*. *rodentium*, WSD-feeding resulted in a modest change in microbial genes involved in metabolism of carbohydrates and vitamins (S7F Fig). More extensive differences were observed 42d post-inoculation with *C*. *rodentium* with differences extending to glycan biosynthesis and lipid metabolism (S7F Fig). Such changes differed between mice that had, or had not, cleared *C*. *rodentium*, including a reduction vs. increase in carbohydrate metabolism genes in WSD-fed mice that had, or had not cleared *C*. *rodentium*, respectively (S7F Fig). While discerning the importance of these differences will require further investigation, we speculate that, collectively, they may contribute to whether *C*. *rodentium* is outcompeted from the gut lumen. While such competition would likely be complex and require many different bacterial species, the marked reduction in Proteobacteria in WSD-fed mice with persistent *C*. *rodentium*, relative to those that had cleared it on either diet (Figs 5F and S7G), suggested that members of this phyla, which *C*. *rodentium* belongs to, might play a role in competing it from the gut lumen. As an initial attempt to explore this possibility, WSD-fed mice with persistent *C*. *rodentium* were intragastrically administered a strain of *Rodentibacter pneumotropicus*, a common, readily culturable murine Proteobacteria species, which resulted in a transient reduction in levels of *C*. *rodentium* (S7H Fig) thus supporting the concept that expansion of other Proteobacteria might play a role in clearing *C*. *rodentium* from the gut lumen.

**Fig 6:**
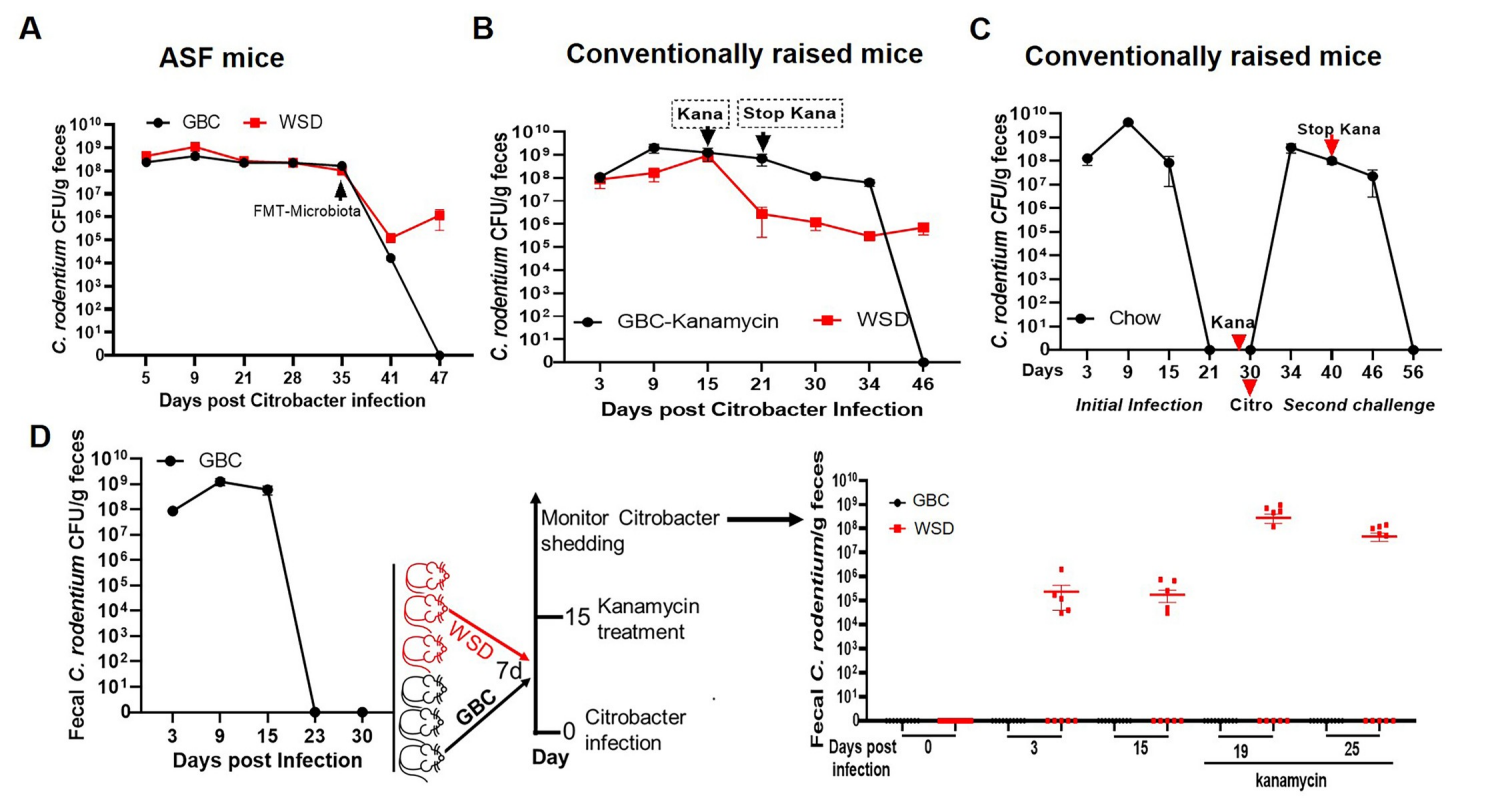
A complex microbiota nourished by GBC mediates clearance of *C*. *rodentium* persistent infection. **A**. ASF mice fed GBC or WSD were orally inoculated with *C*. *rodentium* and 35d later, administered feces from conventional GBC-fed mice. **B.** Conventional GBC-fed mice were orally inoculated with *C*. *rodentium*. 15 days later, mice were left untreated or orally administered kanamycin (1000mg/kg) on days 15, 17, 19, and 21. **C**. GBC-fed, *C*. *rodentium* infected mice were administered kanamycin at day 29 post-inoculation, and re-administered *C*. *rodentium* at day 30, kanamycin was administered every other day until day 40. **D**. GBC-fed mice were administered *C*. *rodentium*, which they uniformly cleared by 30d, at which time they were maintained on GBC or switched to WSD. One week later, mice were re-challenged with *C*. *rodentium* and treated with kanamycin 15 d later. All data are fecal C. *rodentium* CFU on indicated day in that experiment. Data are the means +/- SEM (n = 3–10 mice per group).

To further investigate the notion that microbiota mediates dietary impacts on *C*. *rodentium* clearance, we utilized gnotobiotic mice with a very limited microbiota, specifically the eight bacterial species known as the Altered Schaedler flora (ASF). Unlike germfree mice, such ASF mice have relatively normal immune and metabolic functioning [29]. We observed that, irrespective of whether they were fed GBC or WSD, ASF mice were unable to clear *C*. *rodentium* infection (Fig 6A). Administration of feces from conventional GBC-fed mice resulted in complete clearance of the pathogen in recipients fed GBC, but only led to a moderate and transient lowering of *C*. *rodentium* levels in those fed WSD (Fig 6A). This result suggests that a properly nourished complex microbiota promotes clearance of *C*. *rodentium* from the intestinal tract, analogous to a previous study that demonstrated that complex microbiota was required to mediates clearance of *Salmonella* [30]. To further test this possibility, we sought to mimic the broad ablation of microbiota that results from WSD via a 1-week course of kanamycin in GBC-fed mice, administered 15 days following inoculation with *C*. *rodentium*, at which time rapid clearance would typically commence. Kanamycin delayed clearance of *C*. *rodentium* until 2–3 weeks after its cessation (Fig 6B). Furthermore, administering kanamycin to GBC-fed mice following initial clearance of *C*. *rodentium* infection made those mice prone to re-infection by this pathogen, which was only cleared after its cessation (Fig 6C). Analogous results were obtained in secondary challenge studies. Specifically, while mice maintained continuously on GBC were impervious to re-infection when re-challenged by *C*. *rodentium*, those switched to WSD 7d following its initial clearance were prone to re-infection. Levels of *C*. *rodentium* in these mice were markedly augmented by kanamycin administered 15 days post-inoculation of *C*. *rodentium* (Fig 6D). Together, these results argue that persistence of *C*. *rodentium*, and proneness to re-infection, in WSD-fed mice likely results from their alterations in gut microbiota.

### WSD supplemented with fermentable fiber inulin promotes *C*. *rodentium* initial colonization

**Fig 7:**
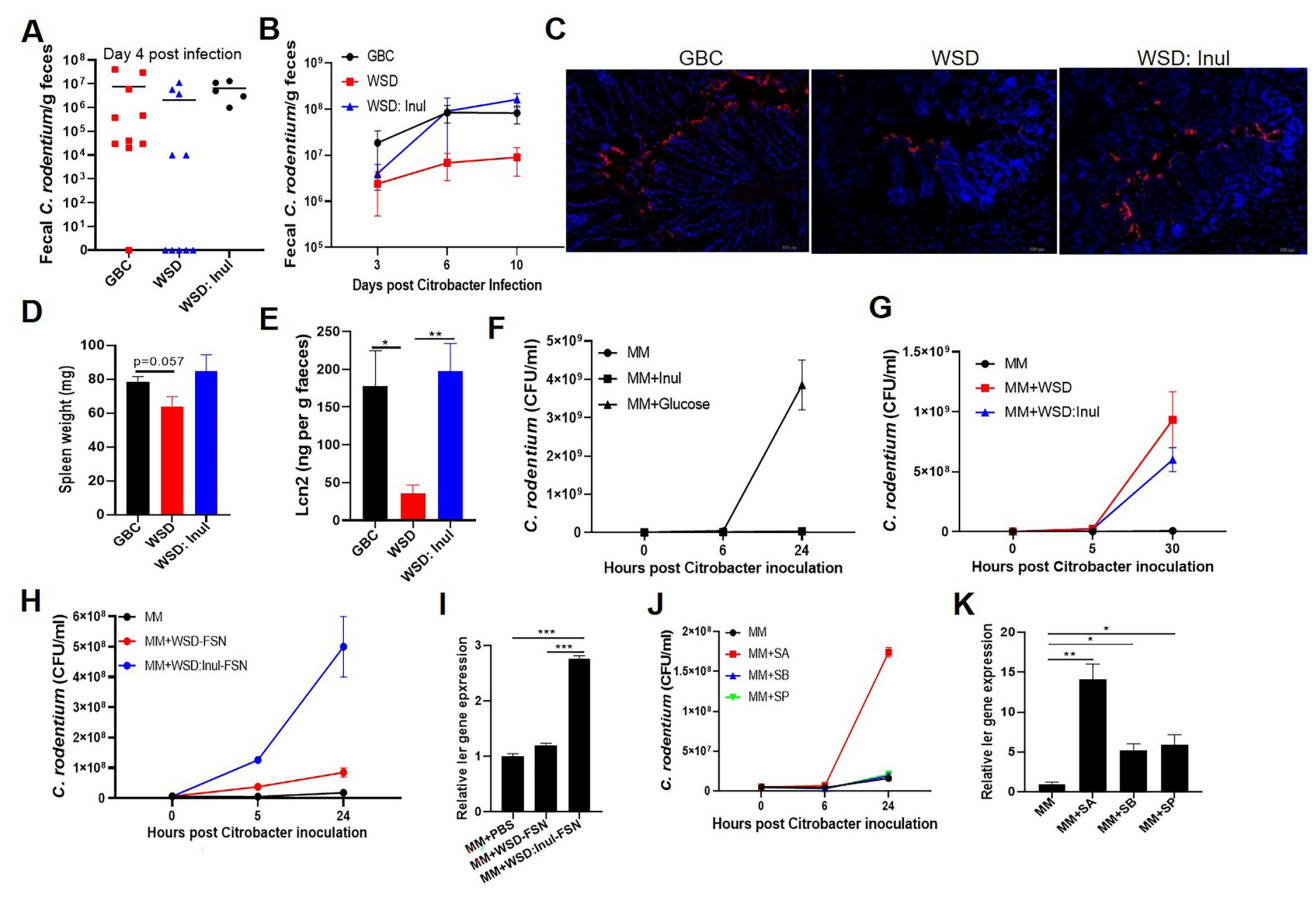
Fermentable dietary fiber promotes initial colonization of *C*. *rodentium*. **A**. Mice fed with indicated diets (n = 5–10) were orally administered 2×10^8^ CFU *C*. *rodentium* per mouse and fecal *C*. *rodentium* CFU measured 4 days later. **B.** Mice were administered with a dose of 4×10^*8*^ CFU *C*. *rodentium /mouse*, fecal *C*. *rodentium* CFU measured at indicated times. **C-E**. Mice were euthanized on day 10 post-inoculation. Immunofluorescent staining of *C*. *rodentium* in colon (C), Spleen weight (D), Fecal Lcn-2 (E). **F-k.** In vitro growth of *C*. *rodentium* in minimal medium supplemented with inulin (Inul) or glucose (Glu) (F), WSD diets +/- inulin (G), fecal supernatant from mice fed WSD +/- inulin (H&I), or short-chain fatty acid including sodium acetate (SA), sodium butyrate (SB) and sodium propionate (SP) (J&K). Expression of ler in minimal media supplemented with fecal supernatant from WSD diets +/- inulin (I) or indicated purified short-chain fatty acid (K) measured by using the reporter ler-lux *C*. *rodentium* strain.

We next turned our attention to early events in *C*. *rodentium* infection, specifically investigating why, despite their low-microbiota density, which one would presume should lower colonization resistance, WSD-fed mice tended to show reduced colonization and inflammation shortly following initial exposure to the pathogen (Figs 2 and 7A). We first probed a role for mucosal immunity, in particular, considering that WSD impacts IL22, IL-18, and adaptive immunity, all of which impede *C*. *rodentium* infection. However, WSD’s reduction of initial *C*. *rodentium* colonization was not impacted by genetic deficiency of IL-22, IL-18, or adaptive immunity (Rag1^-/-^) thus arguing against a role for these host factors (S8 Fig). That *C*. *rodentium* colonized WSD- and GBC-fed similarly in ASF conditions or following antibiotics (Figs 3E and 6A) suggested bacteria or their metabolites present in GBC-fed mice might promote *C*. *rodentium* colonization. In accord with this possibility, enriching WSD with inulin, which restores microbiota density but not composition[17], restored *C*. *rodentium’*s ability to colonize the colon and induce inflammation (Fig 7A–7E). Considering this result and our observation of that fecal supernatant (FSN) from mice fed GBC but not WSD drove microbiota-dependent *ler* expression *in vitro* (Fig 3H), we reasoned that one possible explanation for this might be that *C*. *rodentium* might directly metabolize this fermentable fiber. Yet, in contrast to glucose (positive control), adding inulin to minimal media did not support *C*. *rodentium* growth (Fig 7F) nor enhance the ability of WSD to support *C*. *rodentium* growth in vitro (Fig 7G) thus arguing against this possibility. However, relative to FSN from WSD-fed mice, FSN from mice fed inulin-enriched WSD robustly drove *C*. *rodentium* growth and *ler* expression in vitro (Fig 7H and 7I) thus indicating that the colon environment that resulted from enriching diet with fermentable fiber might support *C*. *rodentium* virulence. Short-chain fatty acids (SCFA), namely acetate, butyrate, and propionate, are the major products of fermentation of inulin and are depleted by WSD and fully restored by enriching WSD with inulin [17]. Acetate, and to a much lesser extent, butyrate and propionate, directly supported *C*. *rodentium* growth and induced ler expression *in vitro* (Fig 7J and 7K). Thus, absence of SCFA in WSD-fed mice might explain the reduced *C*. *rodentium* virulence in mice fed this diet.

## Discussion

**Fig 8:**
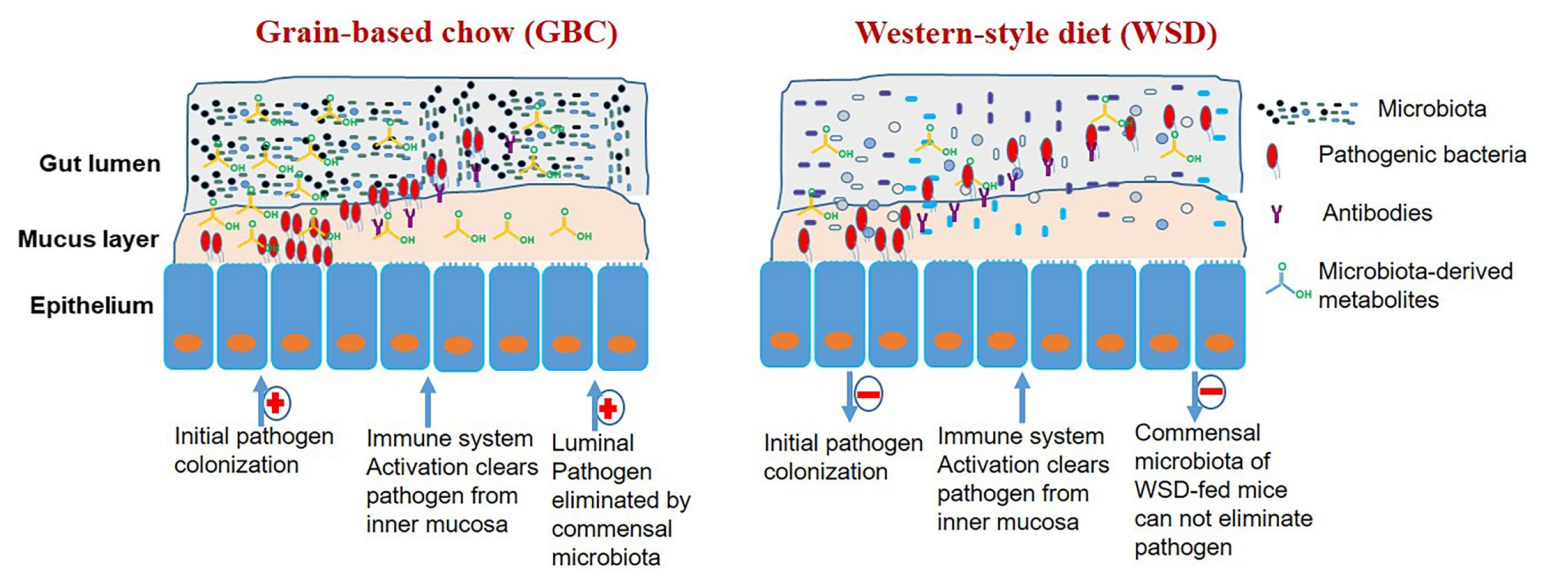
Graphic summary and model. The low-fiber content of WSD impedes initial colonization of pathogens. Immune system functions appropriately irrespective of diet to clear pathogens from the mucosa. Commensal microbiota of mice fed GBC but not WSD is sufficient to eliminate pathogen from the lumen of the intestine. Underlying mechanisms discussed in text.

The importance of the commensal microbiota in determining outcomes that result from exposure to pathogenic bacteria is underscored by the marked elevation in proneness to infection that results from broad-spectrum antibiotics, which typically reduce total gut bacterial loads by orders of magnitude and, moreover, dramatically alter its phylogenic composition [31]. Herein, we report that interactions with bacterial pathogens can also be starkly impacted by the more moderate changes in microbiota that can result from changes in diet (Fig 8). Specifically, we observed that relative to the grain-based chow (GBC) typically consumed by lab mice, feeding “western-style” diet (WSD) reduced initial colonization of attaching-effacing enteropathogenic *E*. *coli*-like gram negative *C*. *rodentium* and decreased its induced acute inflammation. However, WSD-feeding also impaired clearance of this pathogen resulting prolonged infections. Such complex altering the dynamics of this infection suggests that diet may be a key determinant of the heterogenous course/outcomes of such infections and/or the evolving epidemiology of these infectious diseases.

The reduction in initial *C*. *rodentium* colonization that resulted from WSD was, although relatively modest, quite surprising in that this diet results in significant reduction of total gut bacterial loads, which we had presumed might, analogous to antibiotics, enhance *C*. *rodentium* colonization by reducing colonization resistance. Furthermore, our presumption predicted that restoring microbiota density by enriching WSD with inulin would impede *C*. *rodentium* colonization but, in fact, restored it to levels seen in GBC-fed mice. We envisage 2 inter-related mechanisms to explain how dietary fermentable fiber promotes *C*. *rodentium* colonization. Most simply, we postulate that, given that fiber serving as a major microbiota energy source [32], its absence heightens competition for nutrients thus slowing growth of *C*. *rodentium* in the colon. Additionally, the *C*. *rodentium* that is present in the colon of WSD-fed mice displayed lower virulence gene expression, relative to that seen in GBC-fed mice. Moreover, enriching WSD with inulin increases normalized *C*. *rodentium* virulence gene expression. That differences in levels of *C*. *rodentium* and ler expression in mice fed GBC, WSD, and inulin-enriched WSD were recapitulated in vitro by culturing *C*. *rodentium* with bacterial-free fecal supernatants from mice fed these diets, but not the diets directly, suggested a role for bacterial metabolites of these diets, with the major product of fiber fermentation, namely SCFA, being an obvious candidate. Indeed, SCFA, especially acetate, supported *C*. *rodentium* growth and induced virulence gene expression. This result accord with in vitro studies on *Enterohemorrhagic E*. *coli* (EHEC), which also upregulates its virulence gene expression in response to SCFA although the biggest impact was from butyrate and, interestingly, EHEC down-regulates in vitro when exposed to high concentrations of SCFA [33]. A limitation to our study, we hope to overcome in the future, is that in vitro experiments using minimal media were done in aerobic conditions. Nonetheless, we speculate that our observations reflect different bacteria using common gut metabolites as various environmental cues.

While the immune system eliminates *C*. *rodentium* from the epithelium and inner mucus, clearing it from the gut lumen is thought to result from it being outcompeted for nutrients by commensal bacteria [9]. Accordingly, we found that inability of WSD-fed mice to clear *C*. *rodentium* was not associated with defective immune responses and, further, that the persisting pathogen was located in the intestinal lumen. We initially presumed that WSD-induced reduction in microbiota density would be paramount in explaining persistence of *C*. *rodentium* in WSD-fed mice. However, that reducing fat content of WSD facilitated *C*. *rodentium* clearance while enriching GBC with fat delayed it, in both cases without altering microbiota density suggested a role for dietary lipid irrespective of microbiota density. Furthermore, enriching WSD with inulin did not correct the impaired clearance of *C*. *rodentium*, nor proneness to re-infection, further arguing against a role for microbiota density in mediating these aspects of the WSD-induced phenotype and rather suggests a key role for specific taxa or a diverse microbiota in general. The notion that a complex microbiota is required to clear *C*. *rodentium* from the lumen is in accord with our result that ASF mice, which are relatively immunologically and metabolically normal [29], could not clear *C*. *rodentium*, even when fed GBC. Furthermore, clearing *C*. *rodentium* from these mice required not only that a complex microbiota be added but that it be nourished with GBC. Such complexity notwithstanding, our data support a role for expansion of other Proteobacteria in clearing *C*. *rodentium*.

Analogous to results from Nunez and colleagues attained in germfree and antibiotic treated mice [9, 34], we observed that *C rodentium* that persisted in the lumen in WSD-fed mice did not express its virulence genes nor elicit gut inflammation. Yet, we found such persisting *C*. *rodentium* was still associated with a phenotype, namely that of exacerbating insulin resistance induced by WSD. On the one hand, such insulin resistance can be envisaged to be a compensatory means of defense against persisting *C*. *rodentium*. Indeed, dietary iron induces insulin resistance to result in elevated gut glucose levels that suppress virulent factor expression of *C*. *rodentium*, promoting long term asymptomatic carriage of this pathogen[35]. More generally, we note that some degree of insulin resistance is thought to be a normal compensatory response to some states of immune suppression, notably pregnancy. On the other hand, promotion of insulin resistance by bacteria that persist as a result of WSD might have contributed to the increased societal incidence in type 2 diabetes[36]. Accordingly, we speculate that reshaping gut microbiota by nutrients that promote beneficial bacteria that out-compete pathogens/pathobiont may be a means of broadly promoting health.

## Materials and methods

### Ethics statement

All animal studies were performed at Georgia State University under approved animal protocols (IACUC # A17047).

### Mice, Diet and *C*. *rodentium* infection

C57BL/6 Wild type (WT) and IL-18 KO were purchased from Jackson laboratory, and housed at Georgia State University. IL-22 KO mice, generated by Genentech, were bred and housed at Georgia State University. Rag1 KO were purchased from Jackson laboratory and housed in Helicobacter negative and SPF environment. These mice were fed with grain-based chow (GBC) (cat# 5001, LabDiet), or different compositional defined diets (CDD) (S1 Table, Research Diets, Inc), and housed at Georgia State University under approved animal protocols (IACUC # A17047). Mice at 6–8 weeks old age were fed with GBC or specific CDDs for 1 week before orally gavage with 4–6 ×10^8^ CFU/per mouse *C*. *rodentium* strain ICC180, which are bioluminescent and kanamycin resistant. After infection, the feces were collected at indicated time points and resuspend in PBS at concentration of 100 mg/ml. After homogenization, the suspension was serial diluted, then 10 μl of 10-fold serial dilutions was plated on LB agar plate with 100 μg/mL kanamycin. After 21 days post infection, mice with clearance of *C*. *rodentium* was confirmed, then re-infected with *C*. *rodentium* with 4–6 ×10^8^ CFU/per mouse at indicated time points, or GBC fed mice were switched to fed WSD for 1 week before re-infected with *C*. *rodentium* at dose of 4×10^8^ CFU/per mouse. For *Rodentibacter pneumotropicus*, WSD fed mice with chronic infection with *C*. *rodentium* on day 35 post-infection were orally gavaged with 1 ×10^8^ CFU/per mouse *Rodentibacter pneumotropicus [37]*. *C*. *rodentium* fecal was monitored thereafter. For antibiotic treatment: GBC fed mice were infected with *C*. *rodentium* as described above. Some of these mice were gavaged with Kanamycin (1,000 mg/kg in water) every other day from 14 days post infection until indicated time points. Other mice were orally gavaged with kanamycin at day 29 post infection, then infected with *C*. *rodentium* with a dose of 4–6 ×10^8^ CFU/per mouse next day. The mice were euthanized, colon length and spleen weight were measured at the end of experiment. Samples were collected and stored in formalin or -80°C until further analysis. Data collection are summarized in S1 Data.

### Gnotobiotic mouse experiments

C57BL/6 WT germ free mice were inoculated with ASF feces purchased from ASF Taconic, Inc. (Hudson, NY), which contained the 8 ASF strains, to established Altered Schaedler Flora (ASF) mice as previously described [38]. Eight-week-old ASF male mice were fed with autoclaved chow and irradiated WSD for 1 week before infecting with 4×10^8^ CFU/per mouse *C*. *rodentium* strain ICC180, and placed in isolated ventilated caging system (Isocage, Techniplast, Buguggiate VA, Italy) that prevents exogenous bacterial contamination. Fecal samples were collected at indicated days post infection, and the number of *C*. *rodentium* in feces was counted according to described above. After 35 days, the mice were then removed from isocages and transferred to ABSL2 unit, immediately orally administered 200 μl of the fecal suspension which generated from feces of conventional raised mice, and maintained in a sterile condition (autoclaved cage, food and water).

### Infection with wild-type (WT) *C*. *rodentium* and isogenic strains deficient in Ler (Δ*ler*)

8-week old C57BL/6 mice were maintained on GBC or WSD for 1 week and then administered kanamycin by oral gavage (1,000 mg/kg in water). 16 h later, these mice were inoculated with *C*. *rodentium* WT DBS120 strain or *C*. *rodentium* Δ-Ler mutant (6 x 10^8^/per mouse) by oral gavage. Fecal *C*. *rodentium* CFU was measured as described above. Some mice were euthanized at day 10 post-inoculation enabling collection spleen and colon.

### STZ treatment and infection

For inducing high glycemia, eight-week-old male mice fed with GBC were intraperitoneally (I.P.) injected with 40 mg/kg STZ for 4 consecutive days according to previously described [39]. After two weeks, the mice with 5 h fasting glucose between 230 and 265 mg/dl were orally infected with *C*. *rodentium* strain ICC180 at dose of 6 ×10^8^ CFU/per mouse. Mice injected with Sodium citrate buffer (vehicle) were orally infected with the same dose of *C*. *rodentium* as control. The fecal *C*. *rodentium* in these mice was measured by using serial dilution and plate counting.

### Cytokine analyses by qRT-PCR

Total RNA was isolated from colon, liver and epididymal fat using Trizol according to the manufacturer’s instructions (Invitrogen). The mRNA expression level of IL-6, α-defensin, IL-22 and TNF-α was analyzed by using quantitative RT-PCR (qRT-PCR) according to the Biorad One-Step RT-PCR Kit in a CFX96 apparatus (Bio-Rad, Hercules, CA) with the following primers: TNF-α: CGAGTGACAAGCCTGTAGCC, CATGCCGTTGGCCAGGA; α-defensin: GGTGATCATCAGACCCCAGCATCAGT, AAGAGACTAAAACTGAGGAGCAGC; IL-6: GTGGCTAAGGACCAAGACC, GGTTTGCCGAGTAGACCTCA; IL-22: GTGCTCAACTTCACCCTGGA, TGGATGTTCTGGTCGTCACC; 36B4: TCCAGGCTTTGGGCATCA; CTTTATTCAGCTGCACATCACTCAGA. Relative expression in transcript levels were calculated by normalization of each amplicon to housekeeping gene 36B4.

### HE and Goblet cells staining

Colons were fixed in 10% formalin, transferred 1–6 weeks later to 70% ethanol, embedded in paraffin. Then tissues were sectioned at 5-μm thickness for histological examination by hematoxylin & eosin (H&E) staining. For goblet cells staining, deparaffinized and rehydrated colon section were stained with Periodic acid-Schiff (PAS). Crypt length of colon was measured by using image J software.

### ELISA

Fecal samples were homogenized in PBS at 100mg/ml, then centrifuged at 12,000 rpm for 10 min and the supernatant were stored at −80°C. The concentration of LCN-2 in the supernatant was measured by using a DuoSet Mouse Lipocalin-2/NGAL ELISA (R&D Systems) kit. To measure IgA in feces and IgG in serum, 96-well high binding plates were coated with 100μl of bacterial lysate (10 μg/ml in PBS) overnight at 4°C. The plates were washed with PBST (0.05% Tween 20 in PBS) and blocked with 1% BSA for 1 h at room temperature. The fecal supernatant (1:50) and serum (1:1000) were diluted at 1: 50 and 1: 1000 respectively with 1% BSA before applying to 96 well plate. After incubation for 2 h at room temperature, plates were washed and added with goat anti-mouse IgA (SouthernBiotech, 1: 2000) and sheep anti-mouse IgG (GE Healthcare, 1:2000) HRP-conjugated secondary antibody. The optical density (OD) was read at 450 nm (Versamax microplate reader).

### Gut microbiota analysis

Total bacteria DNA was isolated from fecal samples by using QIAamp DNA Stool Mini Kit (Qiagen). To measure the total fecal bacteria number in the feces, quantitative PCR was conducted to analyze the extracted DNA using QuantiFast SYBR Green PCR kit (Biorad) with universal 16S rRNA primers 8F: 5′-AGAGTTTGATCCTGGCTCAG-3′ and 338R: 5′-CTGCTGCCTCCCGTAGGAGT-3′ to measure total bacteria number. Results are expressed as bacteria number / mg feces according to a standard curve. To analyze gut microbiota composition, 16S rRNA gene amplification was conducted as described in the Illumina 16S Metagenomic Sequencing Library preparation guide. Briefly, the extracted DNA was used to amplify the region V4 of 16S rRNA genes by the following forward and reverse primers: 515FB 5′TCGTCGGCAGCGTCAGATGTGTATAAGAGACAGG TGYCAGCMGCCGCGGTAA-3′; 806RB 5′GTCTCGTGGGCTC GGAGATGTGTATAAGAGACAGGGACTACNVGGGTWTCTAAT-3′; These two primers were designed with overhang Illumina adapters. PCR products of each sample were purified with Ampure XP magnetic purification beads (Agencourt) and then run on the bioanalyzer High Sensitivity Chip to verify the size of the amplicon. A second PCR was performed to attach dual indices and Illumina sequencing adapters using Nextera XT Index kit. Products were then quantified before the DNA pool was generated from the purified products in equimolar ratios. The quantity of pooled products was measured before sequencing on the Illumina MiSeq sequencer (paired end reads, 2 × 250 base pairs) at Georgia Institute of Technology Molecular Evolution Core (Atlanta GA). Following demultiplexing, paired-end reads were quality filtered, denoised, merged and chimera removed using DADA2 plugin in Qiime2. Principal Coordinate Analysis (PCoA) plot based on unweighted UniFrac tables was visualized using Emperor in Qiime2 pipeline. Taxonomy was assigned based on Greengenes 16S rRNA gene database. LEfSe (LDA Effect Size) was used to investigate bacterial members at genus level between groups. Sequencing data were deposited at SRA database under accession number PRJNA704355.

### Prediction of the function of gut microbiota

Based on the above 16s sequencing data, the function of gut microbiota before and after C. rodentium infection was predicted by using the phylogenetic analysis of communities by reconstruction of unobserved states (PICRUST)[40]. KEGG pathway functions were categorized at level 2.

### Immunofluorescence staining

Mouse colons were embedded in OCT after euthanasia. The tissues were sectioned at 4 μm thickness, and fixed with 4% formaldehyde for 30 min at RT. After washing with PBS, the section was blocked with blocking buffer (Zymed Laboratories Inc., San Diego, USA) before incubated with anti-*Citrobacter* antibody (Abcam, ab37056) overnight at 4°C as previously described[41]. The section was then stained with Secondary Fluorescent Antibody. After washing in PBS, the tissues were counterstained with mounting medium containing DAPI.

### Measurement of ler and tir expression and Rag2/Il2rg DKO infection

The fresh feces samples were collected at 10 days and 26 days post infection and processed for extraction of total RNAs by using Qiagen RNeasy kit, according to the manufacturer’s protocol. Quantitative real time RT-PCR (qPCR) for ler and tir was performed using a SYBR green PCR master mix and the Step One Real-time PCR system as described previously[9]. Relative expression of ler and tir genes was determined by normalizing to the total *C*. *rodentium* abundance in feces, which was measured by qPCR using fecal DNA as template. To infect mice, the number of *C*. *rodentium* in fecal suspension was measured, and a certain amount of fresh fecal suspension including1×10^7^ cfu was orally gavaged into Rag2/Il2rg DKO mice, and the shedding of *C*. *rodentium* in Rag2/Il2rg DKO mice was monitored after infection.

### Insulin resistance test

WSD fed wild type mice with or without *C*. *rodentium* strain ICC180 persistent infection were maintained on WSD diet for 42 days or 5 months. To conduct insulin tolerance test, mice were fasted for 5 h, and their weight and baseline blood glucose were measured by using a Nova Max plus Glucose meter, then intraperitoneally (I.P.) injected 0.75 U insulin/kg body weight. The blood glucose levels were measured 30, 60, 90 min after injection. After these mice were euthanized, body weight, epididymal fat weight and liver weight were measured.

### *C*. *rodentium* in vitro culture

Fecal samples were collected from mice fed with WSD and WSD supplemented with inulin, and resuspended in PBS at a concentration of 100 mg/ml overnight at 4°C, then centrifuged at 12000 rpm for 10 min to remove fecal debris and bacteria. 20μl of supernatant was added into 1 ml M9 minimal media (12.8 g/L Sodium phosphate (dibasic) heptahydrate; 6 g/L Monopotassium phosphate; 0.5 g/L Sodium chloride; 1 g/L Ammonium chloride; 0.24g/L Magnesium sulphate, 11.1mg/L Calcium chloride, 100ug/ml kanamycin). This culture medium was inoculated with 1μl LB culture medium containing 1–4×10^6^ CFU *C*. *rodentium* strain ICC180, and incubated at 37°C with shaking (200rpm). To examine the influence of fat on the growth of *C*. *rodentium*, 1 ml of M9 minimal media was added with 20 (50:1) or 100 (10:1) ul of corn oil (Sigma-Aldrich cat # C8267), or oliver oil (buying from supermarket), then 1–4×10^6^ CFU *C*. *rodentium* was inoculated, and incubated at 37°C with shaking (200rpm). Growth assay for inulin as carbon source was carried out by adding 20 μl of 20% inulin (Inulin from chicory, Sigma) or 20% D-Glucose as positive control into 1 ml minimal medium, then this culture medium was inoculated with 1μl LB culture medium containing 1–4×10^6^ CFU *C*. *rodentium*, and incubated at 37°C with shaking (200rpm). WSD and WSD supplemented with inulin were resuspended in PBS at concentration of 100 mg/ml, 20μl of suspension was added into 1 ml M9 minimal culture medium to evaluate its influence on *C*. *rodentium* growth. To test the influence of short chain fatty acid on *C*. *rodentium* growth and virulent gene expression, the minimal cultural medium was supplemented with 10 mM sodium acetate, or sodium butyrate or sodium propionate respectively, 1μl LB culture medium containing 1–4×10^6^ CFU *C*. *rodentium* was added into the medium, and incubated at 37°C with shaking (200rpm). The growth of *C*. *rodentium* in the culture medium was measured by using serial dilution and plate counting, expressed as CFU/ml medium. The expression of ler was measured by using a bioluminescent reporter *C*. *rodentium* strain (*C*. *rodentium* WT w/ler-luciferase reporter), in which the ler promoter was fused to *the* lux*CDABE operon of* Photorhabdus luminescens and expressed as fold changes of relative bioluminescence level which normalized by bacteria cell count. *C*. *rodentium* in vitro culture was performed under aerobic condition in current study.

### Statistical analyses

Statistical significances of results were analyzed by unpaired student t test or ANOVA (analysis of variance). Differences between experimental groups were considered significant at *P ≤ 0.05, **P ≤ 0.01 or *** P ≤ 0.001. n.s, not significance.

## Supporting information

S1 Fig WSD-induced alteration in gut microbiota associate with an inability to clear *C*. *rodentium*. Mice were fed the indicated diet for 1 week prior to *C*. *rodentium* inoculation. Relative abundance of gut microbiota phyla before *C*. *rodentium* infection (A-B). Quantification of fecal *C*. *rodentium* at the indicated time points post initial and secondary administration of *C*. *rodentium* (C).
(TIF)

S2 Fig Periodic acid-Schiff staining goblet cells in the crypt of proximal colon. Colon tissue was collected from GBC and WSD fed mice at different time points after infection and stained using Periodic acid–Schiff method, the goblet cells per crypt were counted.
(TIF)

S3 Fig WSD feeding resulted in reduced virulence gene expression by *C*. *rodentium*. **A-B**. Rag1^-/-^IL2R^-/-^ mice were orally administered fecal suspension containing 1×10^7^ CFU *C*. *rodentium* (FCC) as schematized (A). Quantification of fecal *C*. *rodentium* at day 3 post inoculation (B). C-D. Spleen weight (C) and colon length (D) was measured in GBC and WSD fed mice at day 10 post infection. E. Colon tissue was processed for H&E staining.
(TIF)

S4 Fig Persistence of *C*. *rodentium* was not associated with WSD induced obesity. **A-E.** Body weight (A), epididymal fat weight (B), liver weight (C), colon length (D) and colon weight (E) of WSD fed mice with (WSD-N) or without (WSD-C) *C*. *rodentium* clearance measured at the end of experiment.
(TIF)

S5 Fig Hyperglycemia delays but does not prevent clearance of C. *rodentium*. **A.** 5 h fasting glucose measured in mice fed with GBC or WSD or treated with STZ. **B.** Streptozotocin (STZ) treated mice with 5 h fasting glucose between 230 and 265 mg/dl were subjected to infection with *C*. *rodentium*. Fecal *C*. *rodentium* was monitored.
(TIF)

S6 Fig *C*. *rodentium* persistence despite induced expression of IL-22 and secretion of IgA and IgG. **A**. Relative expression level of IL-22 in colon of mice post *C*. *rodentium* infection. **B-C**. The level of IgA in feces and IgG in serum was measured by ELISA.
(TIF)

S7 Fig *C*. *rodentium* infection induces alterations of intestinal microbiota. **A**. Measure of total fecal bacterial DNA by qPCR relative to time of *C*. *rodentium* administration. **B-C.** The relative abundance of the top 7 genera in GBC (B) and WSD (C) fed mice during *C*. *rodentium* infection. **D-E**. Cladogram showing differentially abundant genera in GBC (D) or WSD (E) fed mice during *C*. *rodentium* infection. **F.** Differences in bacterial metabolism function at KEGG level 2 between groups. **G**. The relative abundance of proteobacteria species in WSD fed mice with (WSD-C) and without (WSD-N) detectable *C*. *rodentium* at day 42 post infection. **H**. WSD fed mice with persistent *C*. *rodentium* infection were orally administered *R*. *pneumotropicus* and fecal *C*. *rodentium* monitored.
(TIF)

S8 Fig GBC vs. WSD differences in *C*. *rodentium* are irrespective of IL-18 and IL-22. IL-18 KO (**A-C**), IL-22 KO (**D-F**) and Rag1 KO (**G-H**) mice were fed with GBC or WSD for 1 week before infected with *C*. *rodentium*. Fecal *C*. *rodentium* was measured at indicated days (A, D&G). Spleens were weighted (B&E) and colon was collected to process for HE staining (C&F) for IL-18 KO and IL-22 KO mice; colons collected from Rag1 KO mice were cut longitudinally to remove feces, washed in PBS completely before bioluminescent imaging (H).
(TIF)

S1 Table Diets composition used in this study. (PDF)

S1 Data The numerical data used in all figures are included. (XLSX)
