# Supplementary figures and images for: Western-style diet impedes colonization and clearance of Citrobacter rodentium
Source: PLoS Pathog. 2021 Apr 5;17(4):e1009497. doi: 10.1371/journal.ppat.1009497 (PMC8049485; doi:10.1371/journal.ppat.1009497)

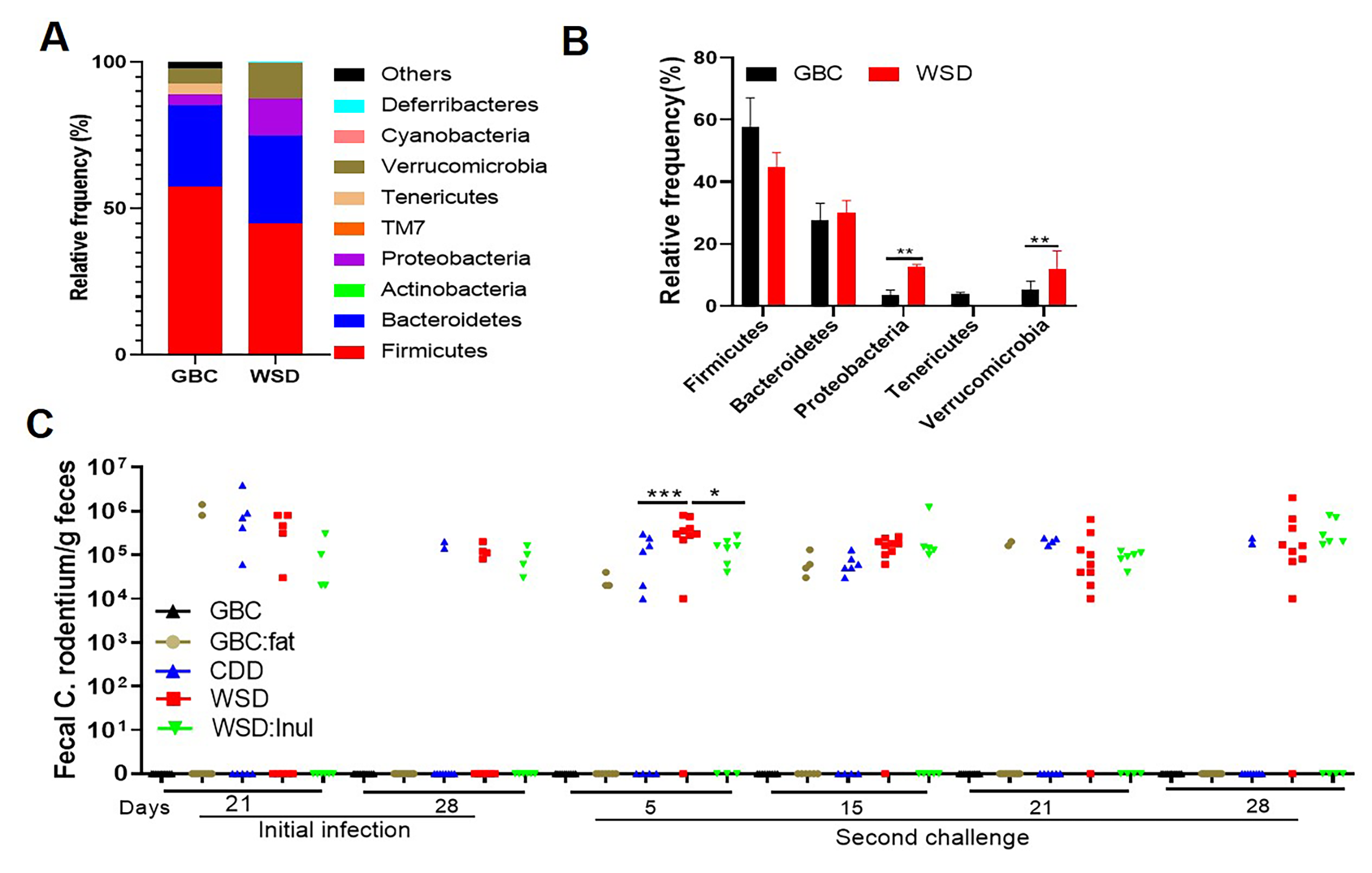

Supplement: S1 Fig — Mice were fed the indicated diet for 1 week prior to C. rodentium inoculation. Relative abundance of gut microbiota phyla before C. rodentium infection (A-B). Quantification of fecal C. rodentium at the indicated time points post initial and secondary administration of C. rodentium (C). (TIF) [file ppat.1009497.s001.tif]

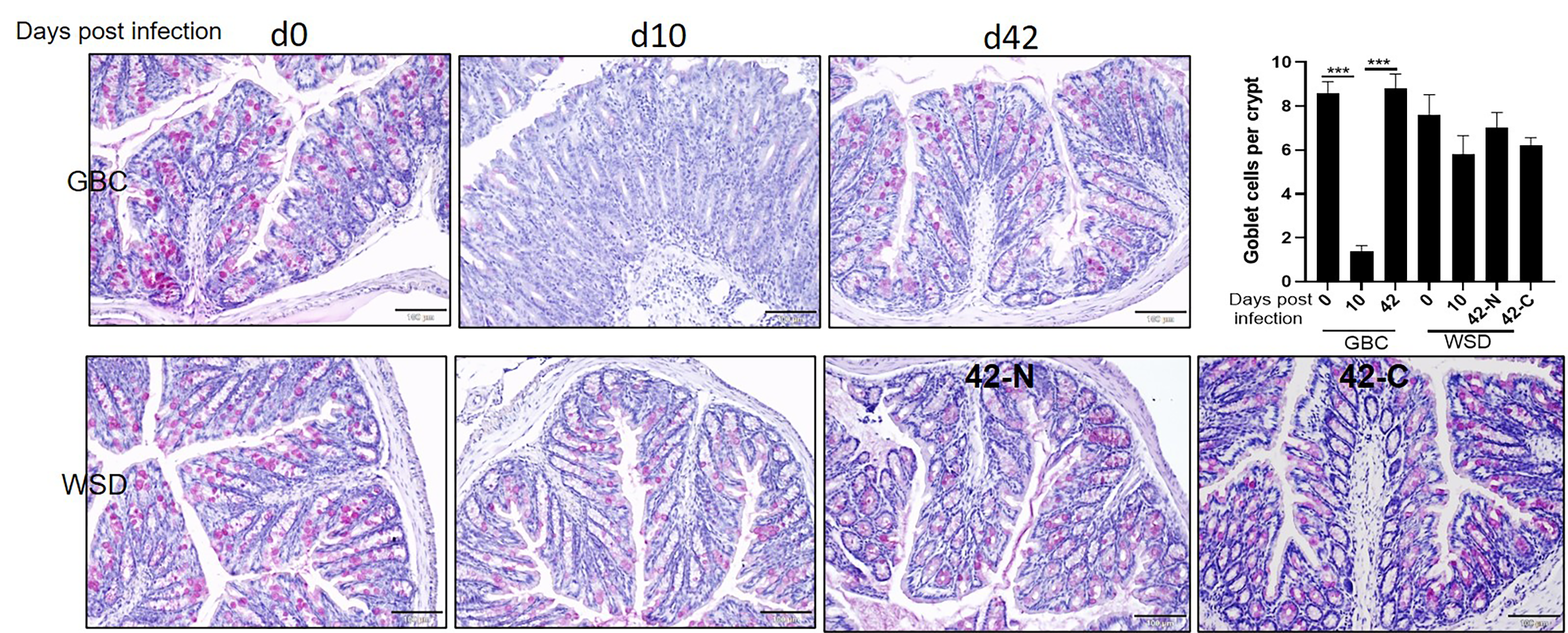

Supplement: S2 Fig — Colon tissue was collected from GBC and WSD fed mice at different time points after infection and stained using Periodic acid–Schiff method, the goblet cells per crypt were counted. (TIF) [file ppat.1009497.s002.tif]

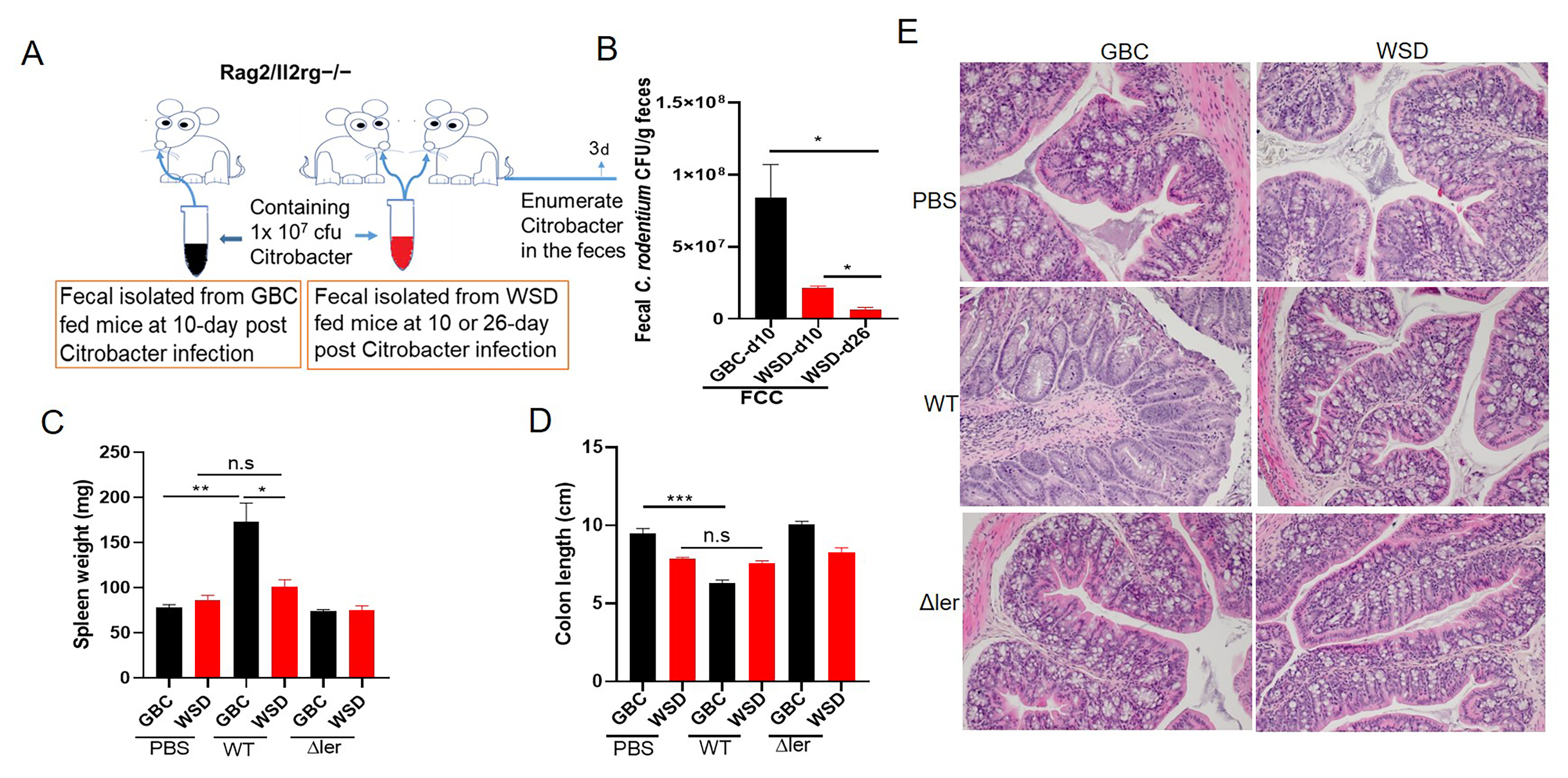

Supplement: S3 Fig — A-B. Rag1-/-IL2R-/- mice were orally administered fecal suspension containing 1×10^7^ CFU C. rodentium (FCC) as schematized (A). Quantification of fecal C. rodentium at day 3 post inoculation (B). C-D. Spleen weight (C) and colon length (D) was measured in GBC and WSD fed mice at day 10 post infection. E. Colon tissue was processed for H&E staining. (TIF) [file ppat.1009497.s003.tif]

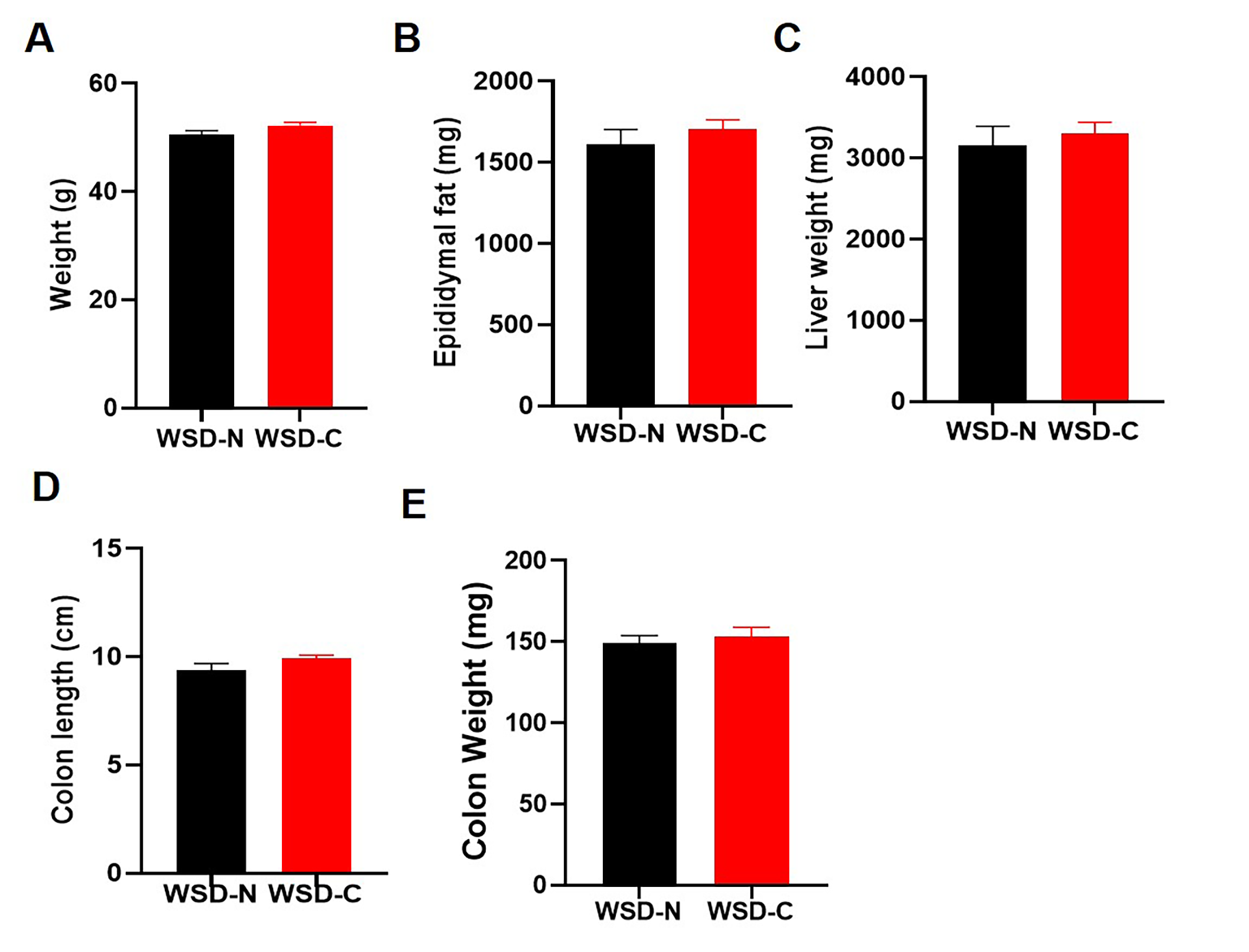

Supplement: S4 Fig — A-E. Body weight (A), epididymal fat weight (B), liver weight (C), colon length (D) and colon weight (E) of WSD fed mice with (WSD-N) or without (WSD-C) C. rodentium clearance measured at the end of experiment. (TIF) [file ppat.1009497.s004.tif]

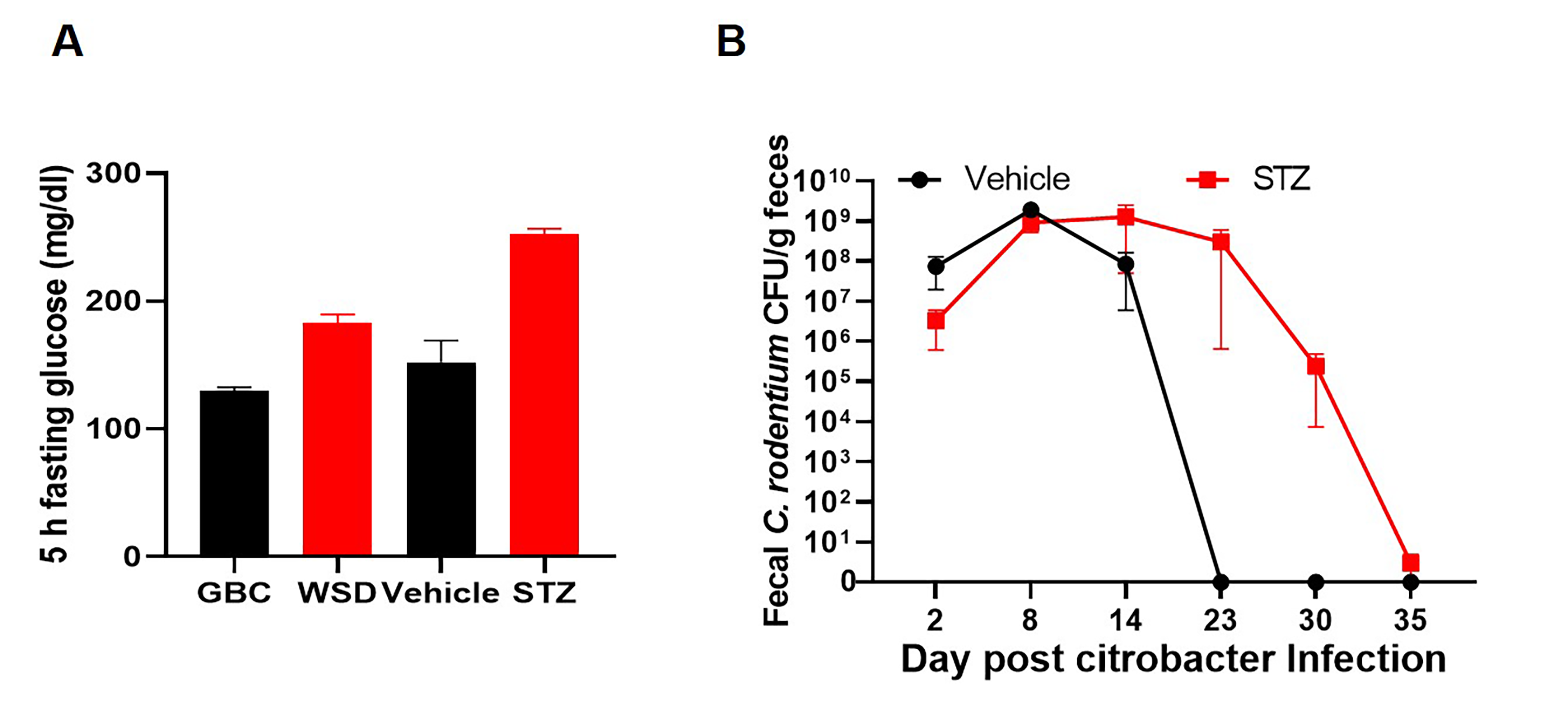

Supplement: S5 Fig — A. 5 h fasting glucose measured in mice fed with GBC or WSD or treated with STZ. B. Streptozotocin (STZ) treated mice with 5 h fasting glucose between 230 and 265 mg/dl were subjected to infection with C. rodentium. Fecal C. rodentium was monitored. (TIF) [file ppat.1009497.s005.tif]

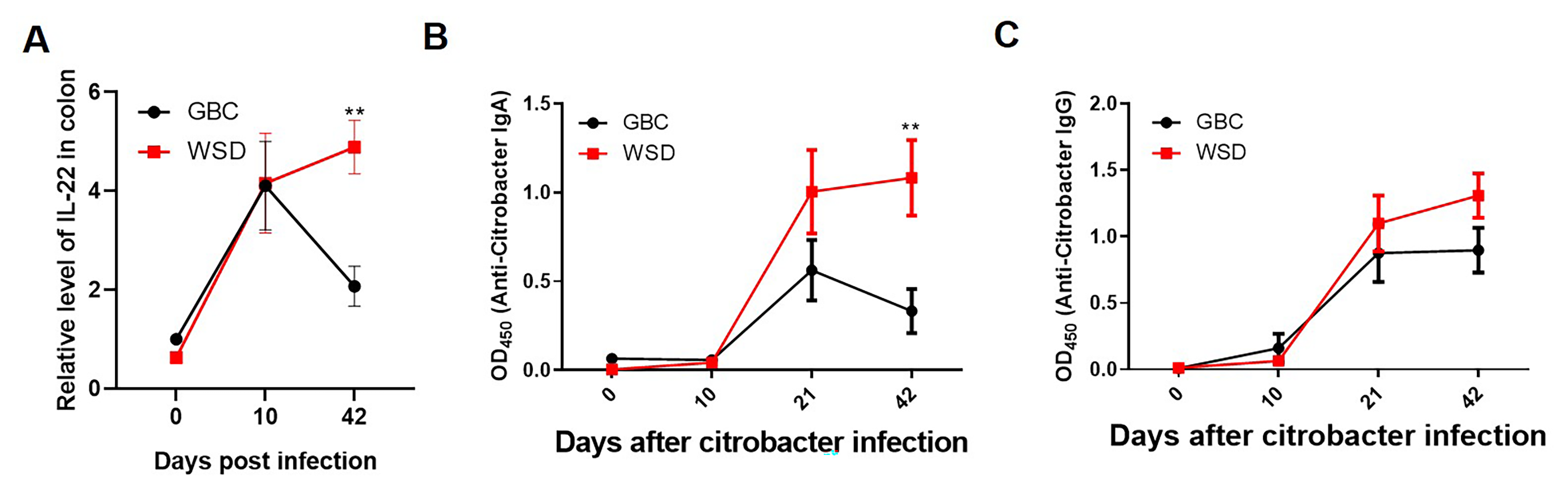

Supplement: S6 Fig — A. Relative expression level of IL-22 in colon of mice post C. rodentium infection. B-C. The level of IgA in feces and IgG in serum was measured by ELISA. (TIF) [file ppat.1009497.s006.tif]

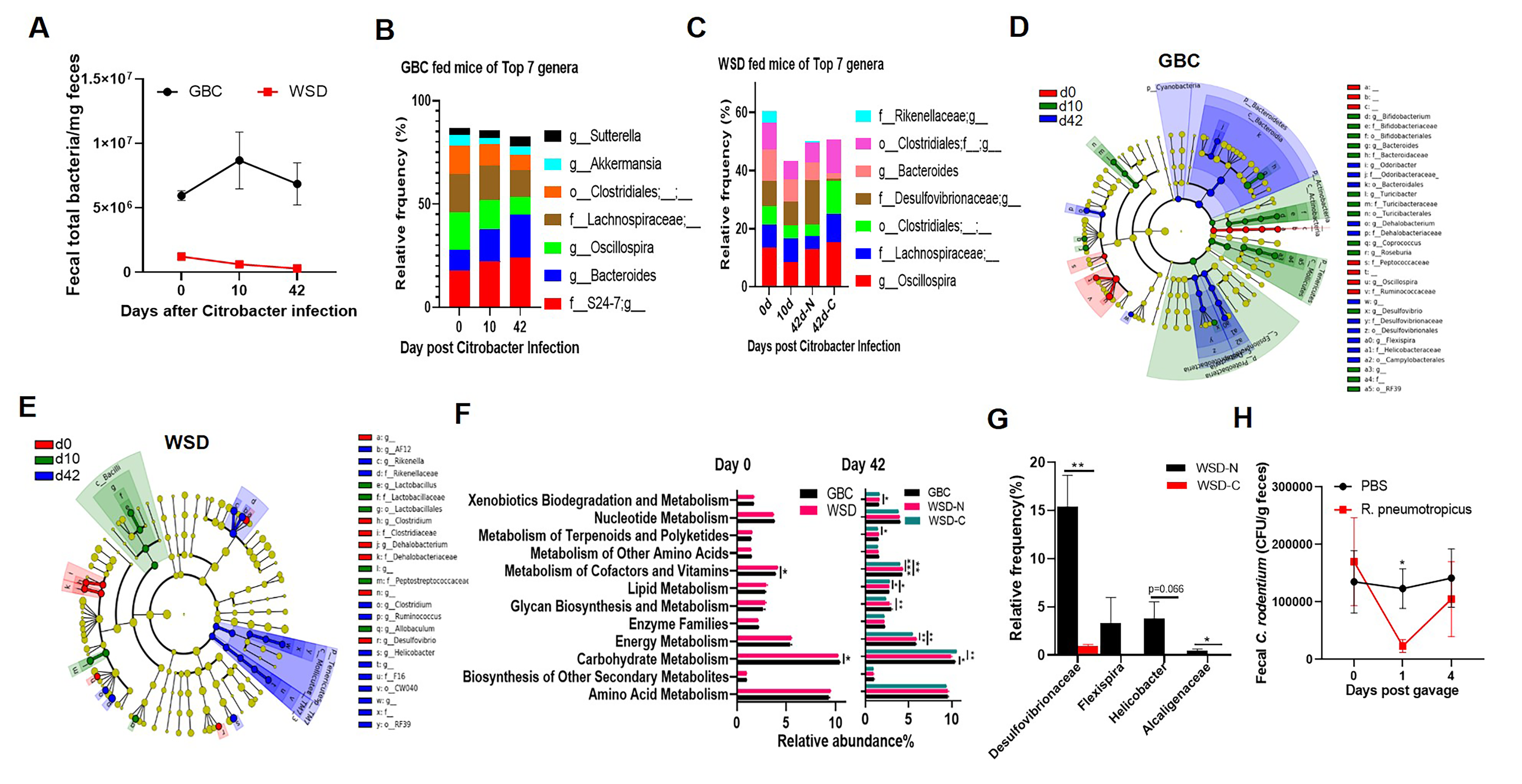

Supplement: S7 Fig — A. Measure of total fecal bacterial DNA by qPCR relative to time of C. rodentium administration. B-C. The relative abundance of the top 7 genera in GBC (B) and WSD (C) fed mice during C. rodentium infection. D-E. Cladogram showing differentially abundant genera in GBC (D) or WSD (E) fed mice during C. rodentium infection. F. Differences in bacterial metabolism function at KEGG level 2 between groups. G. The relative abundance of proteobacteria species in WSD fed mice with (WSD-C) and without (WSD-N) detectable C. rodentium at day 42 post infection. H. WSD fed mice with persistent C. rodentium infection were orally administered R. pneumotropicus and fecal C. rodentium monitored. (TIF) [file ppat.1009497.s007.tif]

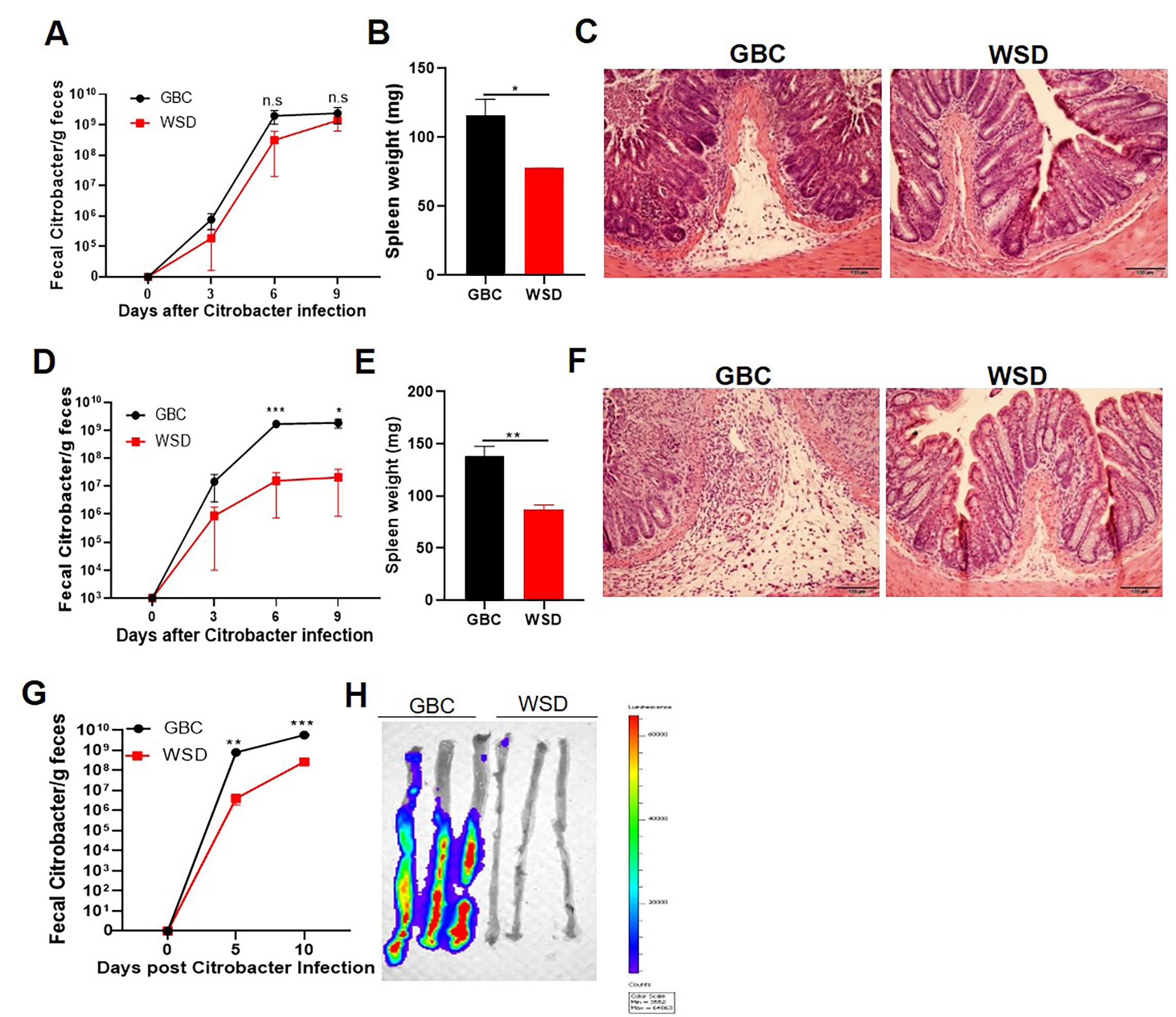

Supplement: S8 Fig — IL-18 KO (A-C), IL-22 KO (D-F) and Rag1 KO (G-H) mice were fed with GBC or WSD for 1 week before infected with C. rodentium. Fecal C. rodentium was measured at indicated days (A, D&G). Spleens were weighted (B&E) and colon was collected to process for HE staining (C&F) for IL-18 KO and IL-22 KO mice; colons collected from Rag1 KO mice were cut longitudinally to remove feces, washed in PBS completely before bioluminescent imaging (H). (TIF) [file ppat.1009497.s008.tif]
