# Supplementary material for: Western-style diet impedes colonization and clearance of Citrobacter rodentium
Source: PLoS Pathog. 2021 Apr 5;17(4):e1009497. doi: 10.1371/journal.ppat.1009497 (PMC8049485; doi:10.1371/journal.ppat.1009497)
Supplement: S1 Table — (PDF) [file ppat.1009497.s009.pdf]

Supplemental table 1. The composition of the purified diets used in this study.

Product #	D12492		D13081106		D12450J	
	WSD		*WSD: Inul*		*CDD*	
	gm%	*kcal%*	gm%	*kcal%*	*gm%*	*kcal%*
Protein	26	*20*	23	*20*	*19*	*20*
Carbohydrate	26	*20*	40	*20*	*67*	*70*
Fat	35	*60*	31	*60*	*4*	*10*
Total		*100*		*100*		*100*
kcal/gm	5.2		4.6		3.8	
Ingredient	gm	*kcal*	gm	*kcal*	gm	kcal
Casein	200	*800*	200	*800*	*200*	*800*
L-Cystine	3	*12*	3	*12*	*3*	*12*
Corn Starch	0	*0*	0	*0*	*506.2*	*2025*
Maltodextrin 10	125	*500*	75	*300*	*125*	*500*
Sucrose	68.8	*275*	68.8	*275*	*68.8*	*275*
Cellulose, BW200	50	*0*	0	*0*	50	0
Inulin, Orafti HP	0	*0*	200	*200*	0	0
Soybean Oil	25	*225*	25	*225*	*25*	*225*
Lard	245	*2205*	245	*2205*	*20*	*180*
Mineral Mix, S10026	10	*0*	10	*0*	*10*	*0*
DiCalcium Phosphate	13	*0*	13	*0*	*13*	*0*
Calcium Carbonate	5.5	*0*	5.5	*0*	*5.5*	*0*
Potassium Citrate, 1 H2O	16.5	*0*	16.5	*0*	16.5	0
Vitamin Mix, V10001	10	*40*	10	*40*	10	40
Choline Bitartrate	2	*0*	2	*0*	2	0
FD&C Yellow Dye #5	0	*0*	0	*0*	0.04	0
FD&C Red Dye #40	0	*0*	0.05	*0*	0	0
FD&C Blue Dye #1	0.05	*0*	0	*0*	0.01	0
Total	773.85	*4057*	873.85	*4057*	1055.1	4057
